# Research on the Mechanism of High-Performance Aluminum Alloy Casting of Frozen Sand Mold Coupled with Negative Pressure

**DOI:** 10.3390/ma19184017

**Published:** 2026-09-21

**Authors:** Lei Luo, Can Luo, Qi Lv, Xiao Liang, Liang Wang, Yanqing Su, Jingjie Guo, Fei Mi, Chao Chen, Binbin Wang

**Affiliations:** 1 Zhengzhou Advanced Research Institute, Harbin Institute of Technology, Zhengzhou 450000, China; luolei51jiayou@163.com (L.L.);; 2 School of Materials Science and Technology, Nanjing University of Aeronautics and Astronautics, Nanjing 210016, China; 3 Shandong Laboratory of Aluminum Advanced Manufacturing in Binzhou, Weiqiao-University of Chinese Academy of Sciences Science and Technology Park, Binzhou 256606, China; 4 Binzhou Institute of Technology, Weiqiao-University of Chinese Academy of Sciences Science and Technology Park, Binzhou 256606, China; 5 Xinxiang Aviation Industry (Group) Co., Ltd., Xinxiang 453000, China; 6 State Key Laboratory of Precision Manufacturing for Extreme Service Performance, Central South University, Changsha 410083, China

**Keywords:** frozen sand mold, negative pressure casting, aluminum alloy, interfacial heat transfer mechanism

## Abstract

Aluminum–copper alloys, typified by ZL205A (AlCu5MnTiCdV, GB/T 1173), pose severe casting challenges due to their wide solidification interval, poor fluidity, and susceptibility to gas porosity and shrinkage defects. This study introduces a negative-pressure frozen sand mold casting process as a solution to these challenges and systematically quantifies its thermal and microstructural advantages over conventional resin sand and atmospheric frozen sand casting. Using inverse heat conduction analysis of multi-point thermocouple data, the interfacial heat transfer coefficient (IHTC) was quantitatively determined for 14 experimental conditions varying in mold type, pressure level, moisture content (2–6 wt.%), and initial freezing temperature (−20 to −40 °C). The results demonstrate that negative pressure combined with frozen sand molds (6 wt.% moisture, −40 °C) produces the highest active-period average IHTC of 236 W/(m^2^·K), representing a 2.8-fold increase over atmospheric frozen sand casting and a 1.6-fold increase over negative-pressure resin sand casting. This enhanced thermal driving force promotes grain refinement and effective gas removal, yielding superior mechanical properties: tensile strength of 200.6 MPa and elongation of 9.3% in the as-cast state, and 478.5 MPa and 7.4% after T6 heat treatment. Using the optimized process parameters, a thin-walled cabin component (Ø180 mm × 300 mm, minimum wall thickness 3 mm) was successfully fabricated without defects. These findings establish quantitative process–thermal–microstructure–property relationships for negative-pressure frozen sand casting of wide-solidification-interval aluminum alloys.

## 1. Introduction

Cast aluminum alloys are extensively utilized in aerospace equipment, primarily serving as structural components for airframes, propulsion systems, and control systems where lightweight and high-strength properties are critical. The most widely used alloys in aerospace manufacturing are the ZL1×× and ZL2×× series, which account for over 95% of the total consumption. These alloys are typically formed using sand casting, permanent mold casting, and investment casting, often combined with special casting techniques such as low-pressure and electromagnetic casting to achieve high-performance aluminum alloy castings. However, aluminum alloys are highly prone to gas absorption and oxidation at elevated temperatures, leading to numerous solidification defects in cast products and reduced yield rates. Meanwhile, with advancements in technology, industrial production demands increasingly stringent requirements for the preparation, processing, and performance of aluminum alloys. In this context, it has become a pressing issue and focus to real-time regulate the solidification behavior of alloys with wide solidification intervals during the solidification process, more effectively refine their microstructure, eliminate structural defects, and enhance mechanical properties. This will further enable near-net shaping of such alloys, reduce post-casting processing, minimize material waste, lower costs, and improve material utilization and production efficiency, while ensuring compliance with performance requirements.

In the 1970s, British companies Booth and British Oxygen Company [1] applied cryogenic technology to casting and invented the frozen mold casting process. The obtained mold was frozen at low temperatures to achieve a certain strength, and the resulting sand mold was essentially identical to those used in traditional sand casting. Moore et al. [2,3] investigated the variation laws of permeability in frozen sand molds and found that coarser sand particles resulted in better final permeability of the mold. The Nagoya Industrial Technology Research Institute [4,5] studied the dynamic behavior of moisture in molds during freezing to compare the relationship between mold temperature and strength. They also discovered that using ferrosilicon powder as an auxiliary binder for frozen casting resulted in minimal deformation. Additionally, they developed a compaction infusion method that reduces cryogen consumption and enables uniform consolidation of the mold. In 2008, S. Tada [6,7] studied factors affecting the compressive strength of frozen sand molds. By using silica colloidal solutions instead of aqueous solutions to produce frozen sand molds, it was found that the molds retained a certain compressive strength, even in a thawed state. In 2015, Shan et al. [8] proposed a digital maskless cutting forming method for frozen sand molds. This method uses pure water as a binder for sand mold casting, where sand particles are mixed with an appropriate amount of water and frozen in a low-temperature environment to form a frozen sand blank. The frozen sand mold is then machined into shape using digital maskless cutting forming equipment based on subtractive manufacturing principles. Liang et al. [9] employed a thermal probe method to measure the thermal conductivity of frozen sand molds with different water contents at various temperatures, establishing a volume-mixing model for estimating the thermal conductivity of frozen sand molds. Current research on frozen mold casting technology primarily focuses on the forming process and comparing the microstructures and properties of simple-shaped castings. However, there is relatively little work addressing the key challenges associated with different alloy systems in the frozen mold casting process. Most experimental samples are based on aluminum–silicon series alloys, while studies on alloys with poor fluidity and high susceptibility to solidification defects, such as aluminum–copper series alloys, remain scarce.

Current research in the field of negative-pressure-assisted casting, both domestically and internationally, primarily focuses on two techniques: V-process casting and lost foam casting. A domestic foundry company [10] adopted high-flow rapid pouring to achieve uniform solidification, ultimately determining key process parameters such as a pouring vacuum level of −0.04 MPa, a holding vacuum of −0.03 MPa, a holding time of 15 min, and a casting insulation time of 8 h. This approach fundamentally resolved issues like cold shuts and hot tearing in the castings. Senthil et al. [11] conducted comparative studies on the solidification process and behavior of alloys under different casting methods. They found that V-process casting effectively eliminates major drawbacks associated with traditional sand casting and die casting, while enhancing the alloy’s filling capacity and improving the surface finish of castings. Gao et al. [12] addressed common defects such as mold collapse and mold wall expansion in lost foam casting of large and medium-sized semi-enclosed castings. They analyzed the production stages where these defects occur and their root causes, subsequently formulating corresponding improvement measures. Practical results demonstrated that tilting the upper plane of semi-enclosed components at a 45° angle during mold filling facilitates better sand filling and compaction. Additionally, installing external vacuum tubes and applying vacuum both internally and externally during pouring ensures balanced negative pressure inside and outside the mold cavity, thereby improving the qualification rate of castings. Zhang et al. [13] analyzed the structure of a tractor transmission housing and the process adaptability of lost foam casting. Through systematic research and development, they successfully established a lost foam casting process for the housing, which holds significant importance for reducing overall manufacturing costs. Liang et al. [14] investigated defects such as sand burning, gas porosity, and sand washing that easily occur in lost foam casting of products like flywheel housings. By adjusting the assembly process of patterns, optimizing the gating system, redesigning the layout of vents and slag traps, and improving the rationality of ingate positions, combined with practical production conditions, he proposed measures to address casting defects in related products. Current research on lost foam casting and V-process casting mainly concentrates on mold-making processes and the control of process parameters. These methods offer advantages such as improved production efficiency, enhanced casting accuracy, and reduced costs to some extent. However, several challenging issues remain unresolved, limiting their broader application.

Since the 1950s, researchers have recognized that the interfacial heat transfer coefficient (IHTC) significantly influences the temperature field evolution during metal solidification, thereby affecting the microstructure and properties of castings. Oukach et al. [15] positioned thermocouples at fixed locations within the casting and mold to record temperature–time curves. Based on these data, they calculated the temperature gradient at the casting/mold interface and derived the variation of the IHTC over time using the formula *h* = *q*/(*T_is_* − *T_im_*), marking the first experimental determination of the IHTC. Han and Singh et al. [16,17,18,19] integrated computer technology with metal solidification processes, employing the Beck inverse algorithm to analyze solidification mechanisms and investigate the temporal variation of the IHTC at the mold/casting interface. Motoyama et al. [20] and Woodbury et al. [21] studied the IHTC at the interface between sand molds and aluminum alloy castings, obtaining its values through different computational methods. In recent years, domestic scholars have made significant progress in the field of IHTC determination. Xu et al. [22] investigated the influence of process factors such as viscosity on the solidification process and analyzed the relationship between the IHTC and viscosity. Their results indicated that higher viscosity leads to a wider air gap layer at the mold/casting interface, corresponding to a lower IHTC. Numerous research teams have also contributed substantial findings through experimental analyses of how the IHTC evolves during solidification, including Zhu et al. [23], Sui et al. [24], Zhang et al. [25], Wang et al. [26], and Cao et al. [27]. Currently, the inverse calculation of the IHTC has been applied to various casting processes such as sand casting, permanent mold casting, and low-pressure casting. Most recently, Dong et al. [28] determined the IHTC at the frozen sand mold/ZL101 alloy interface by combining the finite difference method with conjugate gradient and particle swarm optimization algorithms, revealing that water contents of 4–5 wt.% give rise to two distinct types of IHTC evolution curves; the same group further developed an inverse heat conduction model to estimate the frozen sand mold-melt IHTC during the solidification of A356, quantifying the effects of initial freezing temperature, water content, and molding sand [29]. Qiu et al. [30] established an inverse model for the IHTC of both the outer mold and the inner core of annular aluminum castings, showing that the casting/core IHTC is always higher than the casting/mold value and is governed mainly by the interfacial gap. In parallel, Shi et al. [31] developed a cryogenic gradient forming technique for frozen sand molds and identified an optimal formulation window (sand size 106–212 μm, water content 4–5 wt.%, freezing temperature below −25 °C) that achieved CT8 dimensional accuracy for flywheel housing castings. However, existing IHTC studies on frozen sand molds are limited to atmospheric solidification of Al-Si alloys; there are still no reported investigations on the influence of a negative-pressure environment on the IHTC and heat transfer behavior during casting, nor on wide-solidification-interval Al-Cu alloys such as ZL205A.

Since the morphology and scale of the solidification microstructure are directly governed by the intensity of metal–mold interfacial heat transfer, which is usually characterized by the IHTC, the quantitative linkage between interfacial heat transfer and solidification structure has attracted increasing attention. Yang et al. [32] investigated sand-cast Al-5Cu-0.38Mg-0.32Mn-0.1Ag alloy cylinder heads and found that position-dependent cooling rates refined the equiaxed grains from 116 μm to 62 μm, with yield strengths varying between 140 and 159 MPa, confirming the dominant role of cooling rate in determining grain size and mechanical performance. Mao et al. [33] demonstrated for Al-7Si-0.6Mg alloys that a higher cooling rate increases the nucleation undercooling and the nucleation rate, thereby refining the grains and simultaneously improving strength and ductility. These results indicate that enhancing interfacial heat transfer accelerates heat extraction, raises the solidification undercooling, refines grains, and suppresses gas porosity, which constitutes the mechanistic basis of the process–thermal–microstructure–property relationships examined in the present work.

Therefore, this study focuses on the ZL205A aluminum alloy, a representative Al-Cu-based alloy characterized by a wide solidification interval, poor fluidity, and numerous solidification challenges. A comparative investigation is conducted employing three distinct forming methods: conventional resin sand casting at atmospheric pressure, frozen sand casting at atmospheric pressure, and frozen sand casting under negative-pressure conditions. Furthermore, for the negative-pressure frozen sand-casting process, a comparative analysis is performed using sand molds with varying water contents and initial freezing temperatures. The aim is to elucidate the solidification behavior and the evolution of microstructure and properties of ZL205A aluminum alloy under different processes and frozen sand mold parameters. The specific objectives of this study are: (1) to quantitatively determine the time-dependent IHTC evolution under different mold/pressure conditions using inverse heat conduction analysis; (2) to establish the mechanistic linkage between IHTC magnitude, solidification rate, grain refinement, and porosity suppression; and (3) to identify the optimal frozen sand mold parameters (moisture content and initial freezing temperature) for negative-pressure casting of ZL205A, enabling high-quality thin-walled component fabrication.

## 2. Experiments

### 2.1. Temperature Acquisition Method

**Figure 1:**
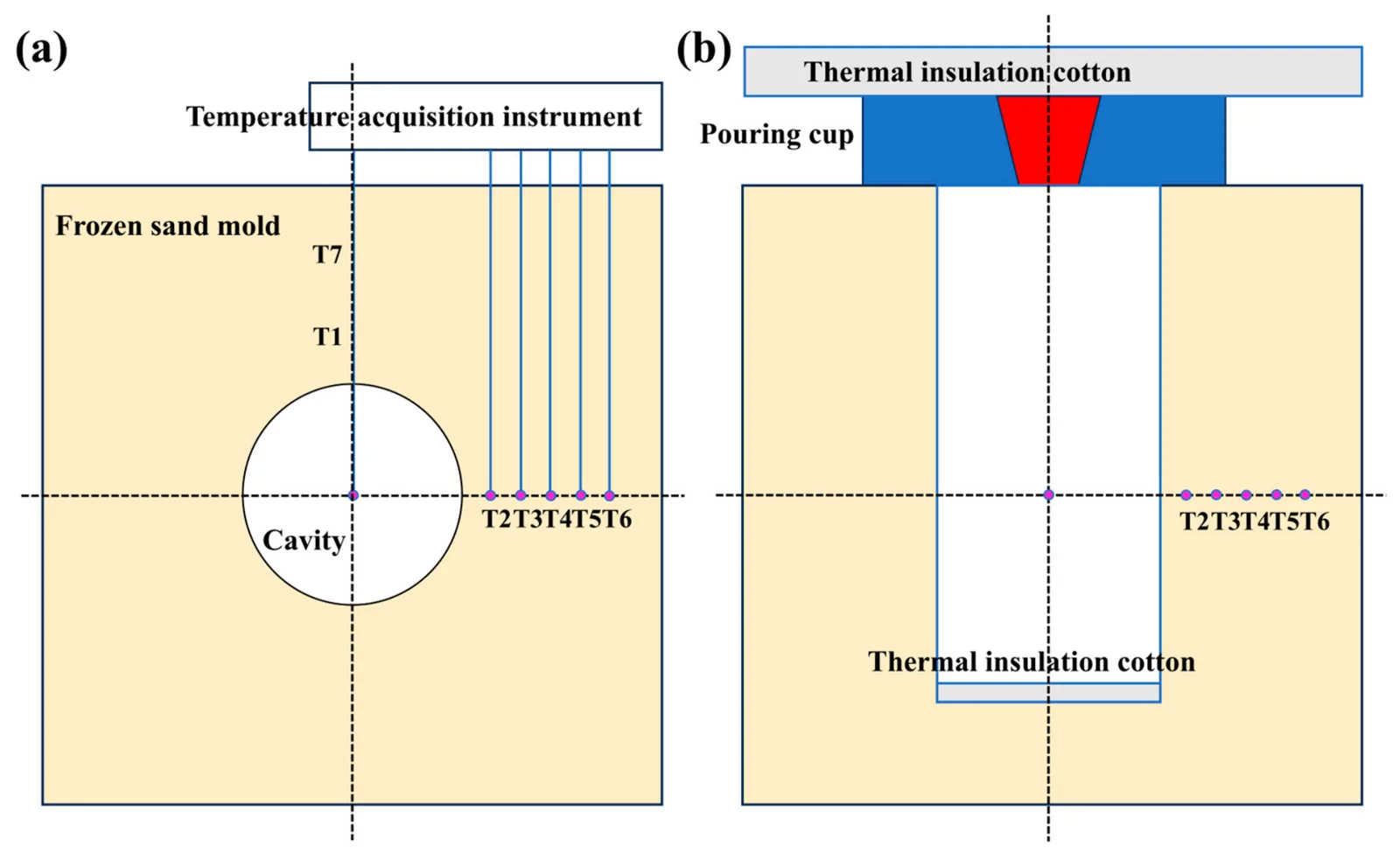
Schematic diagram of temperature collection in frozen sand-mold model. (**a**) Front view of the device. (**b**) Sectional view of the device.

A frozen sand mold with dimensions of 150 mm × 150 mm × 150 mm was designed for temperature acquisition in this study. The mold cavity had a diameter of 40 mm and a height of 100 mm. Temperature data were collected using a multichannel temperature recorder (MIK-4000D, CHN, Hangzhou Meikong Automation Technology Co., Ltd., Hangzhou, China) and seven K-type thermocouples (Hangzhou Meikong Automation Technology Co., Ltd., Hangzhou, China), with a recording frequency of 10 Hz. To ensure accurate temperature measurement of the melt, two thermocouples labeled T1 and T7 were positioned at the center of the melt. Additional thermocouples were placed at distances of 5 mm, 10 mm, 15 mm, 20 mm, and 25 mm from the outer edge of the melt, designated as T2 to T6, respectively, as illustrated in Figure 1. To promote radial heat dissipation from the casting and minimize axial heat loss, thermal insulation cotton was placed at the bottom of the mold cavity. Additionally, a pouring cup and insulation cotton were positioned at the top to provide thermal isolation in the vertical direction.

The K-type thermocouples were calibrated prior to experiments using a precision reference thermometer (accuracy ±0.5 °C) over the temperature range of −50 °C to 900 °C; the expanded measurement uncertainty was ±1.5 °C (k = 2). The thermocouple positions within the mold were confirmed by sectioning a sacrificial mold after freezing to verify that displacement during sand compaction was less than ±1 mm from the nominal positions shown in Figure 1. For negative-pressure-assisted experiments, the pressure–time profile was monitored throughout each experiment using a calibrated pressure gauge (Anhui Zhenglan Instrument Co., Ltd., Chuzhou, Anhui, China; range: 0 to −0.1 MPa, resolution: 0.001 MPa). The vacuum was maintained at −0.03 ± 0.002 MPa throughout the pouring and solidification sequence. The steel shot layer surrounding the mold provided mechanical support and contributed a minor thermal insulation effect; however, as the same steel shot layer configuration was used consistently across all negative-pressure experiments, its contribution to the effective IHTC is embedded in the measured h(t) values and does not affect the relative comparisons between conditions. Nevertheless, because the steel-shot layer and the PE sealing system were present only in the vacuum-assisted configuration, their possible thermal contribution is acknowledged as a limitation of the present study when atmospheric and negative-pressure conditions are compared in absolute terms.

### 2.2. Specimen Preparation and Temperature Measurement Procedures for ZL205A Aluminum Alloy Under Different Processes

A customized mold was used with positioning slots machined at the center of the alloy melt cavity and at distances of 5 mm, 10 mm, 15 mm, 20 mm, and 25 mm from the outer edge of the melt. The alloy used in this study was ZL205A (Chinese standard GB/T 1173 [34]), corresponding to the ISO 3522 [35] designation AlCu5MnTiCdV (Al-5Cu alloy system). The measured chemical composition of the ZL205A alloy used is provided in Table 1.

During the sand mixing process for both frozen sand and resin sand molds, thermocouples were pre-embedded at the designated locations. For the frozen sand molds, after the moist sand was prepared, it was placed in a cold chamber for low-temperature freezing. In this study, resin sand molds and frozen sand molds with different moisture contents and initial freezing temperatures were prepared for temperature measurements under various processes, as summarized in Table 2. An electric resistance melting furnace (Xiangtan Mita Electric Furnace Co., Ltd., Xiangtan, Hunan, China) with a maximum melting temperature of 1200 °C and a capacity of 20 kg was employed for melting the alloy. The furnace was equipped with a temperature monitoring system and a gas inlet at the top. The alloy material was placed in a graphite crucible and melted inside the furnace at a heating temperature of 800 °C to minimize heat loss during transfer to the negative-pressure-assisted casting chamber after removal from the furnace. Once the alloy was fully molten, an inert gas (argon) line was opened for degassing and purification. After degassing, the melt was held for 10 min before pouring. The alloy was poured at a temperature of 740 ± 10 °C and monitored using a calibrated K-type immersion thermocouple. To ensure consistent melt quality across all experimental batches, a standardized degassing procedure was strictly followed: argon was purged through the melt for 10 min at a flow rate of 2 L/min, followed by a 10 min quiescent holding period to allow inclusion settling. While in-line hydrogen measurement (e.g., Telegas) and density-index testing were not performed in the present study, the identical degassing protocol applied to all conditions minimizes inter-batch melt-quality variability. It is also noted that the observed differences in porosity and mechanical properties vary systematically with the studied process variables rather than randomly across batches, which is consistent with the proposed mechanism and indicates that melt-quality fluctuations are unlikely to be the dominant origin of the observed trends. Direct quantification of melt hydrogen content will be carried out in future work to further substantiate this aspect. Upon completion of melt treatment, the sand molds corresponding to different processes were moved to the pouring station. The thermocouples were connected to the temperature acquisition instrument, and final preparations were made for pouring.

For gravity pouring, the resin sand molds and frozen sand molds were positioned in the pouring area. The temperature measurement system was connected, and the thermocouple locations were verified to ensure accuracy. Thermal insulation cotton was placed at the bottom of the mold cavity to minimize heat loss. The molten alloy was then removed from the furnace and poured into the mold. Upon completion of pouring, cotton insulation was also placed on top of the mold to provide vertical thermal isolation. For negative-pressure-assisted pouring, the prepared resin sand and frozen sand molds were placed inside a negative-pressure chamber. Thermal cotton insulation was positioned at the bottom of the mold cavity to inhibit downward heat transfer. The temperature measurement system was connected and calibrated. The surrounding areas of the molds were filled with steel shot until the surface of the shot was level with the top of the molds. A polyethylene (PE) film was used to seal the entire assembly. To prevent molten metal leakage during pouring, which could pose a safety hazard, dry sand was spread over the sealed film. The negative-pressure system was inspected and tested to ensure the safety of all hydraulic and electrical circuits. Following technical specifications for the negative-pressure equipment, the negative pressure was gradually applied at −0.01 MPa to conform the film tightly to the mold surface. After the film was fully sealed, the molten alloy was withdrawn from the furnace and poured into the mold. Prior to pouring, the negative pressure level was adjusted to −0.03 MPa. This procedure allowed the completion of negative-pressure-assisted casting and temperature-measurement experiments for both frozen sand and resin sand molds. Subsequently, the castings underwent a heat treatment process of 510 °C for 7 h, followed by 520 °C for 9 h, and finally 175 °C for 5 h.

Silica sand (SiO_2_ ≥ 99 wt.%) with a mean grain size of 0.18–0.25 mm (AFS grain fineness number: 55–65) was used for all frozen sand molds. Distilled water was added gravimetrically to achieve the target moisture contents of 2, 4, and 6 wt.%, verified by drying a representative sample at 105 °C to constant mass before and after mixing. The compressive strength and permeability of frozen sand molds were not systematically measured in this study; however, qualitative assessment of mold integrity after casting provides indirect evidence that molds with 6 wt.% moisture content and −40 °C initial freezing temperature maintained sufficient structural integrity throughout pouring and solidification, while lower moisture content conditions (2 wt.%) exhibited partial disintegration near the melt–mold interface.

### 2.3. Performance Testing and Analysis of Specimens

The prepared specimens were ground and polished prior to subsequent analysis. The microstructure morphology on both transverse and longitudinal sections, the distribution of chemical elements within the microstructure, the dispersion of precipitated phases, and the fracture surface morphology of the tensile samples were examined using an Electron Probe Microanalyzer (EPMA, JXA-8230, Nippon Electronics, Akishima, Tokyo, Japan). Tensile tests at room temperature were conducted on alloy castings produced under different processes using an electronic universal testing machine (Instron 5569, Instron, Norwood, MA, USA). A minimum of five tensile specimens (*n* = 5) were prepared and tested for each experimental condition. Results are reported as mean ± standard deviation. One-way analysis of variance (ANOVA) was performed to assess the statistical significance of differences in tensile strength and elongation among conditions; pairwise comparisons were conducted using Tukey’s honestly significant difference (HSD) test at a significance level of α = 0.05. The tensile direction was aligned parallel to the temperature gradient direction during solidification, with a strain rate of 10^−3^ s^−1^. The tensile specimens had a gauge length of 20 mm, a gauge diameter of 3 mm, and an overall length of 45 mm.

### 2.4. Calculation Method of Interfacial Heat Transfer Coefficient

The interfacial heat transfer coefficient (IHTC), h(t), between the alloy melt and the sand mold was determined using the standard approach. The heat flux density at the melt–mold interface, *q*(*t*), was first calculated based on the temperature gradient measured at the thermocouple positions within the mold material, using Fourier’s law:

(1) $$q(t)\;=\;-\lambda_{\mathrm{mold}}\;\times \;\frac{\mathrm{dT}}{\mathrm{dx}}/\mathrm{interface}$$

where *λ_mold_* is the effective thermal conductivity of the mold material (resin sand or frozen sand), determined from the temperature-dependent volume-mixing model [9], and dT/dx is the spatial temperature gradient estimated by finite difference from adjacent thermocouple readings.

The IHTC was then calculated as

(2) $$h(t)=q(t)/(T_{\mathrm{is}}(t)-T_{\mathrm{im}}(t))$$

where *T_is_*(*t*) is the interpolated temperature at the inner surface of the sand mold adjacent to the melt, and *T_im_*(*t*) is the corresponding melt surface temperature. Both values were interpolated from the discrete thermocouple data using second-order polynomial fitting in the spatial domain, combined with smoothing in the time domain to reduce noise amplification inherent in the inverse approach.

For frozen sand molds, the effective thermal conductivity *λ_mold_* was treated as temperature- and phase-dependent, transitioning from the frozen state (ice-bonded) value at temperatures below 0 °C to the wet sand value as temperature rises above the melting point of ice. The transition was modeled using a linear interpolation scheme consistent with the latent heat plateau observed in the experimental temperature curves. The calculated IHTC values were validated by verifying that the reconstructed temperature profiles at intermediate thermocouple positions (T2–T6) agreed with experimental measurements within ±5 °C.

## 3. Results and Discussion

### 3.1. Study on Heat-Transfer Behavior

**Figure 2:**
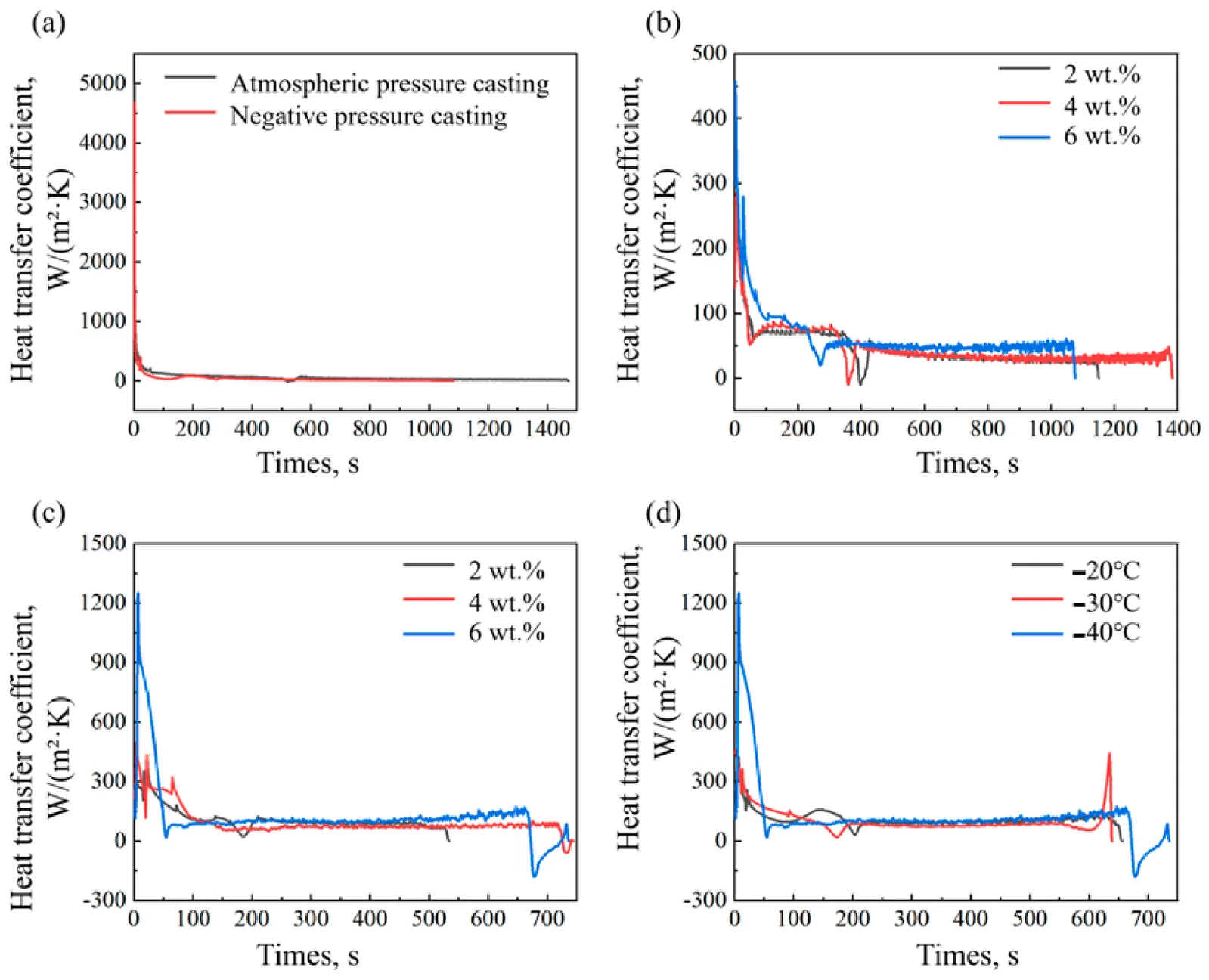
Interfacial heat transfer coefficient under different process parameters. (**a**) IHTC of resin sand mold under atmospheric and negative pressure. (**b**) IHTC of frozen sand molds with different moisture contents under atmospheric casting. (**c**) IHTC of frozen sand molds with different moisture contents at −40 °C under negative-pressure casting. (**d**) IHTC of frozen sand molds with 6 wt.% moisture at different temperatures under negative-pressure casting.

To quantitatively characterize the interfacial heat-transfer behavior, the interfacial heat transfer coefficient (IHTC), *h*(*t*), was calculated for each experimental condition using the inverse heat-conduction method, following the procedure described in Section 2.4. The calculated IHTC values, including peak values and time-averaged values during the active solidification period (750–400 °C), are summarized in Table 3. The temporal evolution of IHTC under representative conditions is shown in Figure 2. Under atmospheric resin sand casting (No. 1), the IHTC reached a peak value of 4969 W/(m^2^·K) immediately upon pouring, corresponding to the initial thermal shock at the melt–mold interface, then rapidly decreased to an average of 151 W/(m^2^·K) during the high-temperature stage (>600 °C) and 82 W/(m^2^·K) over the entire active solidification period. Under negative pressure (No. 2), the peak IHTC was comparable at 4675 W/(m^2^·K); however, the sustained IHTC was marginally higher (87 W/(m^2^·K) average) despite the faster overall temperature rise of the mold, which ultimately reduced the thermal gradient in the later solidification stage. Under frozen sand-mold casting (No. 5, 6 wt.%, −40 °C, atmospheric), the peak IHTC reached 458 W/(m^2^·K), with an active-period average of 84 W/(m^2^·K). The characteristic temperature plateau observed in the cooling curves reflects a period of dynamic thermal equilibrium associated with ice-bridge melting and moisture evaporation, which contributed to sustained heat transfer across the melt–mold interface. With the application of negative pressure to frozen sand molds (No. 8, 6 wt.%, −40 °C, −0.03 MPa), the IHTC increased dramatically: the peak reached 1250 W/(m^2^·K), the average during the early high-temperature stage (melt T > 600 °C) was 837 W/(m^2^·K), and the overall active-period average was 236 W/(m^2^·K), approximately 2.8× higher than frozen sand casting under atmospheric conditions, and 1.6× higher compared to resin sand casting under the same negative pressure. This substantial enhancement is attributed to negative pressure accelerating gas and vapor removal from the melt–mold interface, increasing direct contact area, and establishing a steeper, sustained temperature gradient across the mold thickness.

The characteristic three-stage evolution of the IHTC shown in Figure 2 can be interpreted phenomenologically in terms of the successive states of the melt–mold interface during cooling. In the first stage, immediately after pouring, the liquid metal wets and conforms to the mold surface under the largest instantaneous temperature difference of the whole process, so that the intense thermal shock and the good conformal contact of the liquid metal against the cold mold surface produce the sharp initial increase in the IHTC. In the second stage, as the melt cools below the liquidus, a solidified shell forms at the interface; the shell contracts while it thickens, and an interfacial gap develops accordingly. For the frozen sand molds, this effect is compounded by the melting of the ice bonds near the interface, which absorbs latent heat and releases moisture that forms a vapor-rich interfacial layer. Both the developing gap and the vapor-rich layer increase the interfacial thermal resistance, so that the IHTC decreases and passes through a minimum, which corresponds to the period of most advanced gap growth and shell consolidation, i.e., to the late stage of solidification of the alloy. In the third stage, the interface temperature has stabilized near the plateau values discussed above; the evaporation of the residual moisture and the transport of vapor through the interface reach a quasi-steady balance with the condensation and re-freezing of water in the colder outer regions of the mold. Under this dynamic balance, the interfacial thermal resistance varies only slowly, and the sustained temperature gradient across the mold maintains an approximately constant IHTC until the end of the active cooling period.

The effect of negative pressure on this evolution is also consistent with the curves in Figure 2: the continuous extraction of gas and vapor from the interface keeps the interfacial vapor layer thinner than under atmospheric conditions and limits the growth of the interfacial resistance, which is why the negative-pressure curves settle at a higher quasi-constant IHTC level (e.g., 236 W/(m^2^·K) for No. 8 versus 84 W/(m^2^·K) for No. 5) rather than exhibiting only a higher transient peak. Because interfacial gap widths, vapor generation rates, and in situ vapor pressures were not directly measured in the present study, the above description is an interpretation supported by the observed temperature and IHTC trends rather than a directly demonstrated mechanism.

**Figure 3:**
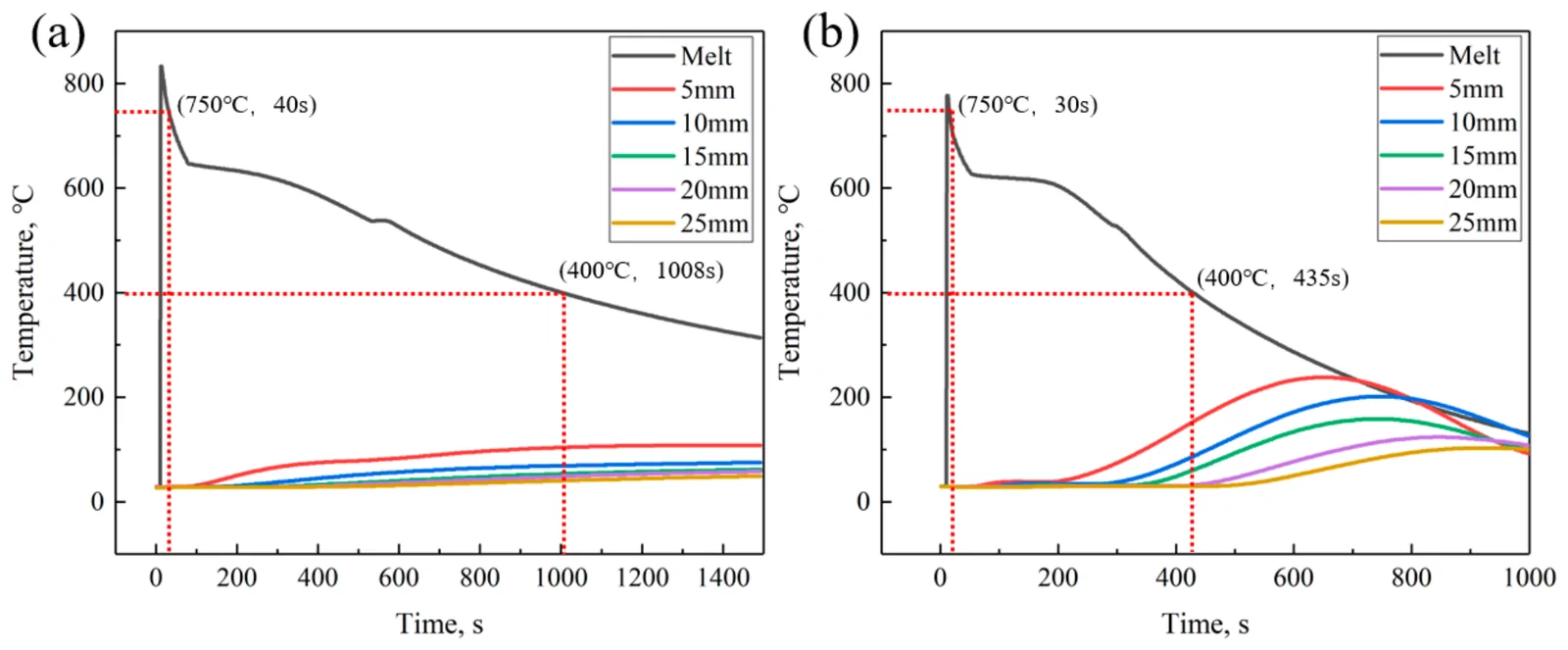
Comparison of temperature curves for different casting processes using resin sand molds. (**a**) Atmospheric-pressure casting. (**b**) Negative-pressure casting.

In the investigation of the temperature profiles of ZL205A aluminum alloy, considering variations in the melt temperature at the moment of pouring, the comparative analysis focused primarily on the cooling process from 750 °C to 400 °C. The temperature curves for the resin sand-mold casting process under both atmospheric and negative-pressure conditions are shown in Figure 3. It can be observed that under atmospheric pressure, the time required for the alloy melt to cool from 750 °C to 400 °C was 968 s, while under negative-pressure conditions, this duration was reduced to 405 s. Furthermore, under atmospheric casting, when the melt temperature decreased to 400 °C, the temperature on the inner surface of the resin sand mold (adjacent to the melt) reached a maximum of 104 °C, while the outer surface registered 40 °C. In contrast, under negative-pressure-assisted casting, the temperature rise of the resin sand mold starting from ambient temperature was more pronounced. The inner surface temperature reached a maximum of 153 °C, while the outer surface remained at 30 °C. These results indicate that negative-pressure-assisted casting significantly enhances heat conduction, increasing the time-averaged IHTC from 82 W/(m^2^·K) (No. 1) to 87 W/(m^2^·K) (No. 2) in resin sand, and from 84 W/(m^2^·K) (No. 5) to 236 W/(m^2^·K) (No. 8) in frozen sand molds, representing a 2.8-fold increase. Under atmospheric conditions, the resin sand mold behaves more like a homogeneous medium, with slow heat transfer occurring between the melt and sand particles, among the sand particles themselves, and between the sand and the ambient air. The application of negative pressure promotes the formation of a steeper temperature gradient within the resin sand mold. In addition, the negative-pressure effect transforms the mold into a thermal bridge between the melt and the external environment, accelerating heat transfer and dissipation across the melt–sand, sand–sand, and sand–air interfaces. Therefore, the application of negative pressure during the casting process effectively increases the solidification rate of the alloy. Moreover, even when the melt experiences the same temperature drop or heat loss, the outer surface of the resin sand mold under negative pressure remains at a relatively low temperature due to the graded thermal distribution induced by the negative pressure.

**Figure 4:**
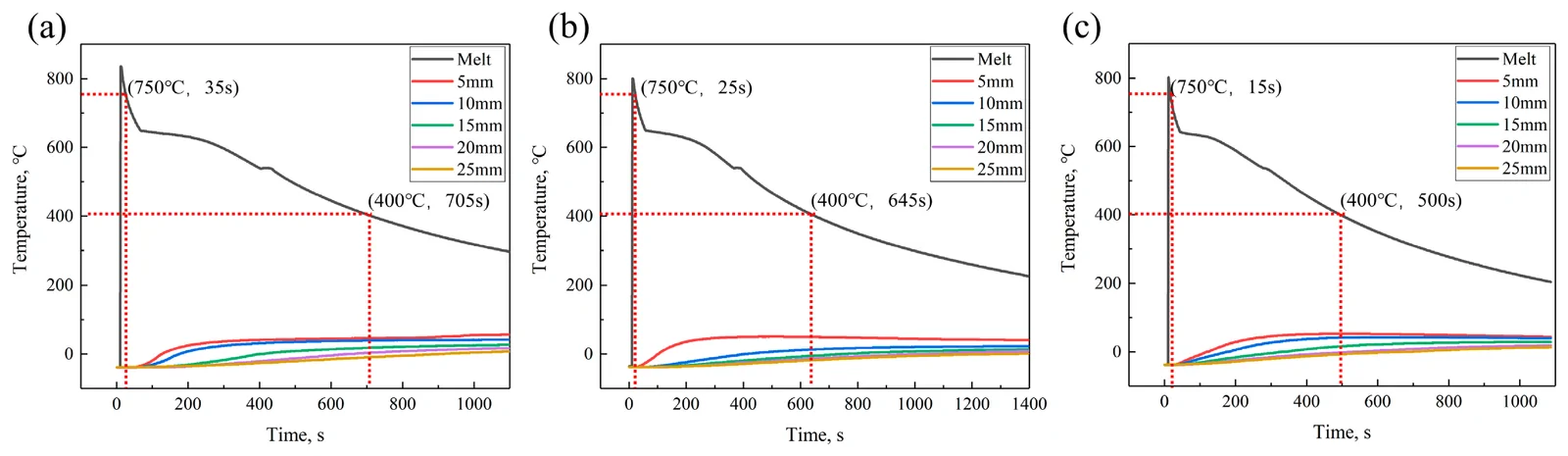
Comparison of temperature measurement curves for atmospheric pressure casting of frozen sand molds with different parameters. (**a**) Water content 2 wt.%, initial freezing temperature −40 °C. (**b**) Water content 4 wt.%, initial freezing temperature −40 °C. (**c**) Water content 6 wt.%, initial freezing temperature −40 °C.

The temperature curves for the casting process using frozen sand molds with different parameters under atmospheric pressure are analyzed in Figure 4. Comparative results indicate that, compared with resin sand casting under atmospheric conditions, the use of frozen sand molds significantly enhances heat conduction and the interfacial heat transfer coefficient (IHTC) between the alloy melt and the mold. As the moisture content of the frozen sand mold increases, the time required for the alloy melt to cool from 750 °C to 400 °C decreases from 670 s (at 2 wt.% moisture content) to 485 s (at 6 wt.% moisture content). Furthermore, during casting with frozen sand molds, the temperature curve of the mold exhibits an initial rise followed by a “stable” plateau as the melt temperature decreases. This phenomenon occurs because, within this plateau range, the ice crystal bonding bridges within the frozen sand mold begin to fracture and transform into liquid water. As the temperature continues to rise, the liquid water starts to evaporate. The rapid evaporation of water induces a “dynamic equilibrium” in the temperature of the frozen sand mold within this region, manifesting as an apparent “stable” plateau.

At a moisture content of 6 wt.%, the plateau temperature observed at the interface between the alloy melt and the frozen sand mold is 53 °C. In comparison, plateau temperatures of 50 °C and 46 °C are recorded at moisture contents of 4 wt.% and 2 wt.%, respectively. This difference is consistent with the interpretation that, during the heating of frozen sand molds with higher moisture content, a region with relatively concentrated water vapor forms between the mold and the alloy melt. Within this localized environment, the relative humidity is high, and the water vapor content in the air approaches or reaches saturation. Under such conditions, the rate at which water molecules escape from the liquid surface into the air decreases. Within this interpretation, a higher temperature is required to sustain continuous evaporation of the liquid water, leading to a higher plateau temperature in frozen sand molds with elevated moisture content.

**Figure 5:**
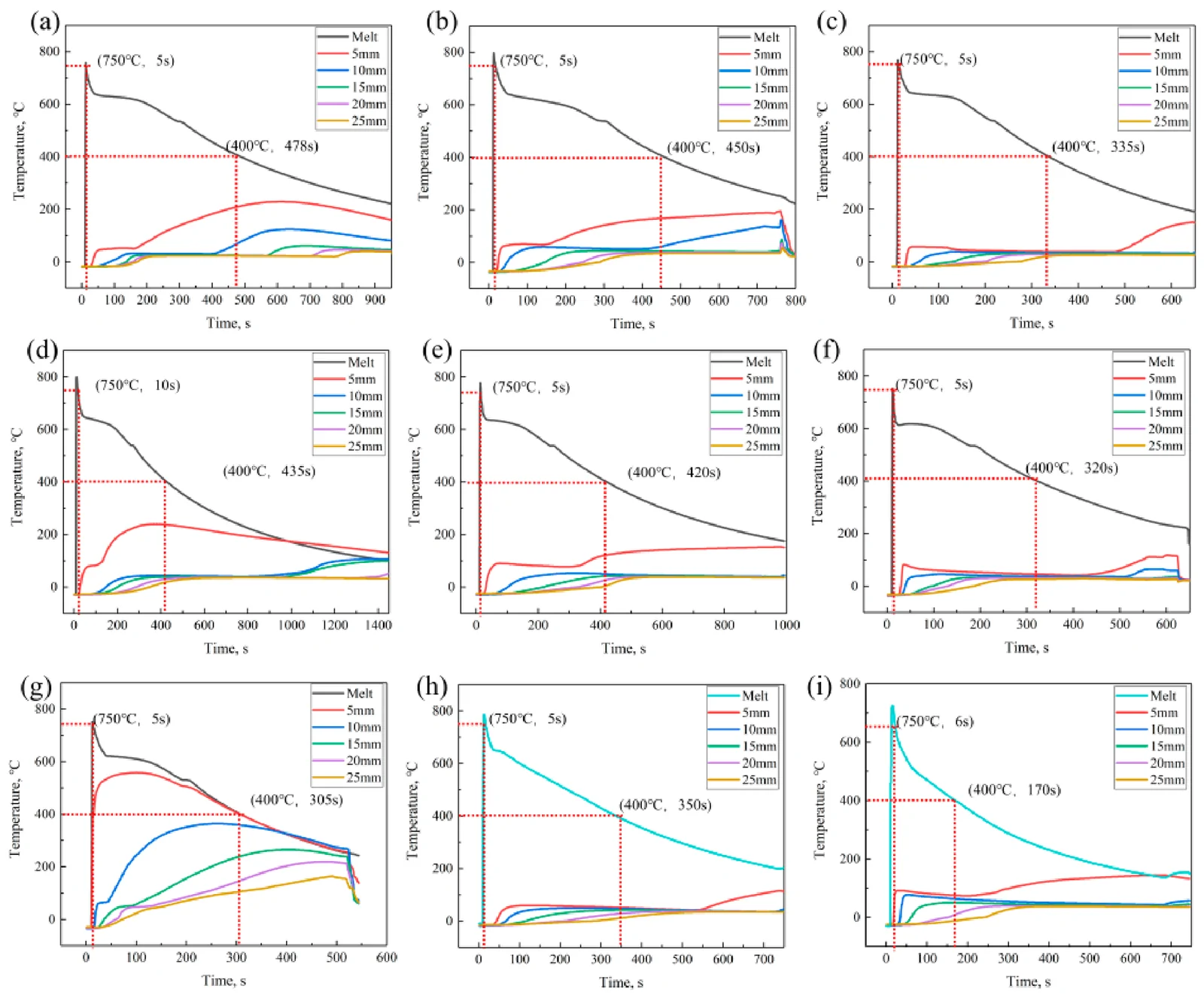
Temperature measurement curves for negative-pressure casting of frozen sand molds with different parameters. (**a**) Water content 2 wt.%, initial freezing temperature −20 °C. (**b**) Water content 4 wt.%, initial freezing temperature −20 °C. (**c**) Water content 6 wt.%, initial freezing temperature −20 °C. (**d**) Water content 2 wt.%, initial freezing temperature −30 °C. (**e**) Moisture content 4 wt.%, initial freezing temperature −30 °C. (**f**) Moisture content 6 wt.%, initial freezing temperature −30 °C. (**g**) Water content 2 wt.%, initial freezing temperature −40 °C. (**h**) Water content 4 wt.%, initial freezing temperature −40 °C. (**i**) Water content 6 wt.%, initial freezing temperature −40 °C.

The temperature curves for the negative-pressure-assisted casting process using frozen sand molds with varying parameters, including moisture contents of 2 wt.%, 4 wt.%, and 6 wt.%, and initial freezing temperatures of −20 °C, −30 °C, and −40 °C, are analyzed in Figure 5. Comparative results reveal that, compared with atmospheric frozen sand casting, the application of negative pressure significantly enhances the cooling rate of the alloy melt, improves the interfacial heat transfer coefficient (IHTC), and increases heat transfer efficiency between the melt and the frozen sand mold. For instance, compared to atmospheric casting using frozen sand molds with moisture contents of 2 wt.%, 4 wt.%, and 6 wt.% and an initial freezing temperature of −40 °C, the introduction of a negative pressure of −0.03 MPa reduced the time required for the alloy melt to cool from 750 °C to 400 °C from 670 s, 620 s, and 485 s to 305 s, 350 s, and 170 s, respectively. In contrast, shortly after the alloy melt is poured into the cavity of the frozen sand mold under negative-pressure conditions, the mold in contact with the melt experiences rapid heating. At lower moisture contents, the negative pressure rapidly removes moisture, leading to conditions where heat is transferred directly between dry sand and the alloy melt, as shown in Figure 5g. Under such circumstances, the characteristic temperature plateau may disappear, often resulting in mold collapse and failure to form a sound casting.

As illustrated in Figure 5, in negative-pressure-assisted frozen sand casting, a lower initial freezing temperature results in a steeper temperature gradient induced by the negative-pressure environment. This enhances the thermal driving force, accelerates interfacial heat transfer, and increases the IHTC between the alloy melt and the frozen sand mold. Consequently, the region of the frozen sand mold closest to the melt experiences a concentrated temperature rise. Regardless of the moisture content, as the initial freezing temperature decreases, the high IHTC causes the entire frozen sand mold to function as an efficient heat-transfer channel with a significant temperature gradient. The lower the initial freezing temperature, the more pronounced this gradient becomes. This results in a faster and higher temperature rise in the mold region contacting the melt, while the outer region remains at a lower temperature. Correspondingly, the plateau temperature formed at the interface increases with decreasing initial freezing temperature.

In summary, as shown in Table 3, compared to resin sand casting (No. 1, h_avg_ = 82 W/(m^2^ K)), frozen sand casting under atmospheric conditions (No. 5) maintained a comparable active-period average IHTC of 84 W/(m^2^ K) but with a notably higher early-stage IHTC of 137 W/(m^2^ K) due to the initial large thermal gradient. The application of negative pressure to frozen sand molds dramatically amplified this effect, with the optimized condition (No. 8) achieving h_avg_ = 236 W/(m^2^ K) and peak h = 1250 W/(m^2^ K). Under negative-pressure conditions, decreasing the initial freezing temperature from −20 °C to −40 °C at 6 wt.% moisture increased the active-period IHTC from 120 (No. 14) to 236 W/(m^2^ K) (No. 8). At a fixed freezing temperature of −40 C, decreasing moisture content from 6 wt.% (No. 8, 236 W/(m^2^ K)) to 4 wt.% (No. 7, 125 W/(m^2^ K)) and 2 wt.% (No. 6, 131 W/(m^2^ K)) resulted in reduced but non-monotonic IHTC, as partial loss of interfacial contact near the melt boundary, presumably associated with local weakening of the low-moisture mold, reduced the effective heat transfer.

The two types of average IHTC values reported in Table 3 were calculated directly from the transient *h*(*t*) curves obtained by the inverse method described in Section 2.4. The early-stage average was computed as the time-averaged value of *h*(*t*) over the initial high-temperature interval in which the measured melt temperature remained above 600 °C, which corresponds to the cooling of the fully liquid alloy immediately after pouring; it therefore reflects the period of best thermal contact and strongest thermal shock at the interface. The active-period average was computed as the time-averaged value of *h*(*t*) over the complete cooling interval from 750 °C to 400 °C, which spans the cooling of the liquid, the solidification of the alloy within its wide freezing range, and the early cooling of the solid; this average therefore also weights the later stages, in which the forming shell, the developing interfacial gap, and the vapor-rich interfacial layer reduce the instantaneous IHTC. For this reason, the active-period average is systematically lower than the early-stage average (e.g., 137 versus 84 W/(m^2^·K) for No. 5, and 837 versus 236 W/(m^2^·K) for No. 8), and the ratio between the two quantities indicates how strongly the interfacial heat transfer degrades as solidification proceeds. The active-period average is the more representative quantity for comparing process conditions because it integrates the interfacial heat transfer over the entire solidification window that governs the microstructure and mechanical properties.

### 3.2. Evolution of Microstructure

**Figure 6:**
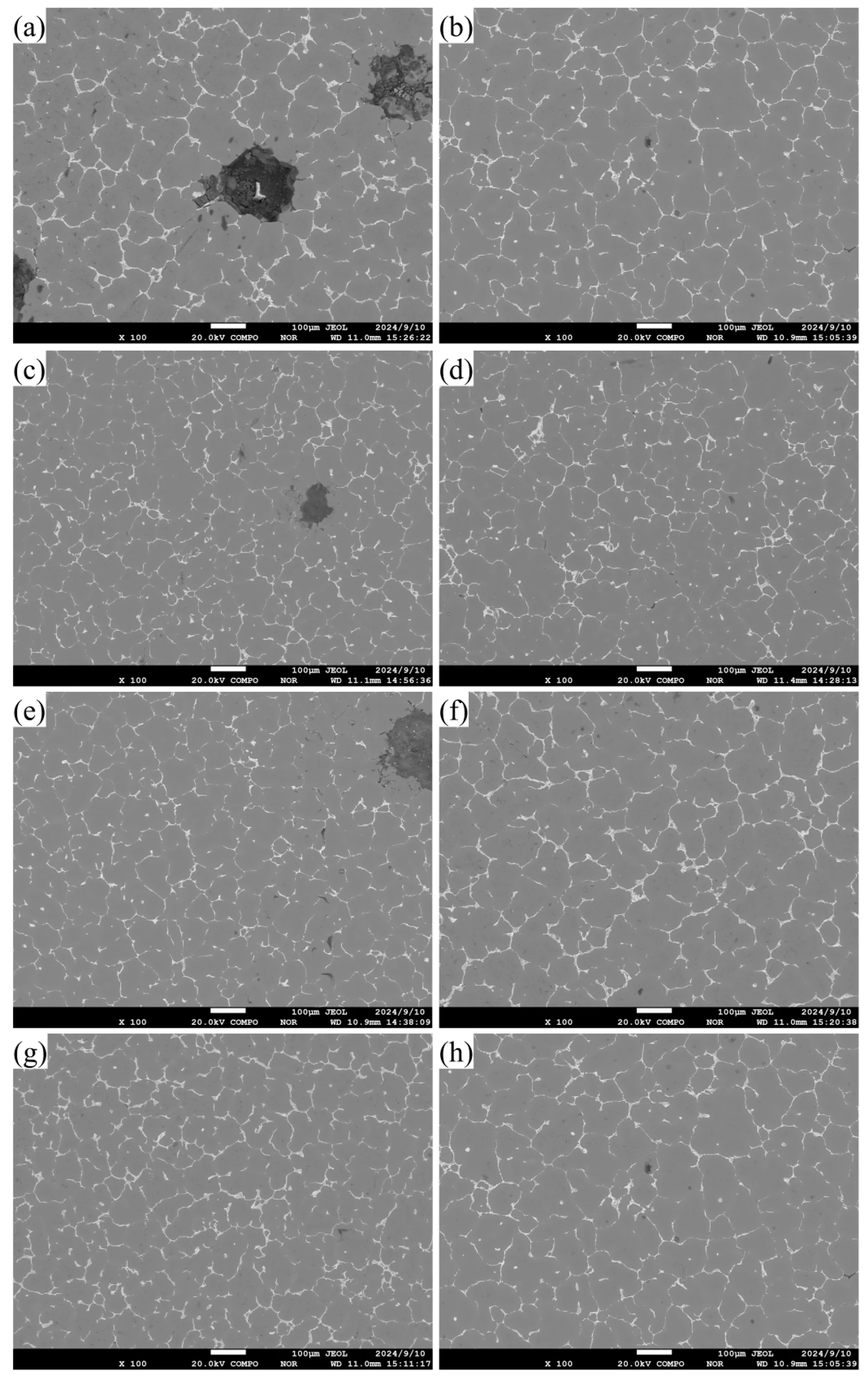
Microstructure of different casting processes. (**a**) Resin sand mold normal pressure casting; (**b**) resin sand mold negative-pressure casting; (**c**) 6 wt.% water content, −40 °C frozen sand mold normal-pressure casting; (**d**) 6 wt.% water content, −40 °C frozen sand mold negative-pressure casting; (**e**) 6 wt.% moisture content, −20 °C frozen sand mold negative-pressure casting; (**f**) 6 wt.% moisture content, −30 °C frozen sand mold negative-pressure casting; (**g**) 4 wt.% moisture content, −40 °C frozen sand mold negative-pressure casting; (**h**) 2 wt.% moisture content, −40 °C frozen sand mold negative-pressure casting.

Quantitative grain size analysis was performed on EPMA images using ImageJ (1.54p) software (linear intercept method, ASTM E112). Calibration was established from the 100 um scale bar in each image. The results (Table 4) show that resin sand casting under atmospheric conditions (Figure 6a) produced the coarsest grain structure (~95 um average), while negative-pressure resin sand casting (Figure 6b) yielded slightly coarser grains (~105 um) despite improved porosity conditions. Frozen sand molds progressively refined the grain structure: atmospheric frozen sand casting at 6 wt.%/−40 °C (Figure 6c) produced grains of ~70 um, while negative-pressure frozen sand casting under the same conditions (Figure 6d) achieved the finest grains of ~58 um, consistent with the highest active-period IHTC of 236 W/(m^2^ K) recorded for this condition (Table 3). Increasing the initial freezing temperature under negative-pressure conditions resulted in coarser grains (~93 um for −20 °C, ~78 um for −30 °C; Figure 6e,f), correlating with the progressively lower early-stage IHTC values (147 and 198 W/(m^2^ K), respectively; Table 3). These quantitative results confirm a monotonic relationship between the active-period IHTC and the resulting grain refinement level.

The microstructure morphology of the alloy samples was examined using an electron probe microanalyzer (EPMA), with the results shown in Figure 6. A clear comparison between the microstructures of castings produced by resin sand casting under atmospheric-pressure (Figure 6a) and negative-pressure conditions (Figure 6b) reveals significant differences. To emphasize the effect of negative pressure, no vent holes were included in the sand molds in this study. As a result, during atmospheric resin sand casting, trapped gases could not escape efficiently, leading to the formation of numerous gas pores. In contrast, the negative-pressure condition facilitated rapid gas extraction, and no significant porosity was observed in the microstructure. However, under negative-pressure-assisted casting, the grain size of the alloy microstructure was coarser compared to that under atmospheric conditions. Although negative pressure enhances the interfacial heat transfer coefficient (IHTC), the rapid heating of the sand mold caused its overall temperature to rise quickly, approaching the temperature range of the cooling alloy melt. This reduced the thermal driving force and consequently decreased heat-transfer efficiency during the later stages of solidification. In atmospheric resin sand casting, the temperature of the mold increased more gradually, helping maintain a consistent thermal gradient and driving force for heat transfer, which ultimately accelerated the overall solidification of the alloy. Therefore, comparatively speaking, atmospheric conditions resulted in more favorable solidification characteristics than negative-pressure conditions in resin sand casting.

Compared to resin sand casting, both atmospheric- and negative-pressure-assisted frozen sand casting produced castings with significantly finer grain structures. When using a frozen sand mold with 6 wt.% moisture content and an initial freezing temperature of −40 °C under atmospheric conditions, the microstructure of the alloy casting was notably refined, as shown in Figure 6c. This refinement is attributed to the low temperature of the frozen sand mold, which rapidly increased the solidification rate of the alloy melt, promoting grain refinement. However, a large amount of water vapor generated at the melt–mold interface could not escape in time, resulting in the formation of numerous fine gas pores in the microstructure.

When negative-pressure-assisted frozen sand casting was employed under the same conditions (6 wt.% moisture, −40 °C initial freezing temperature), as illustrated in Figure 6d, the microstructure exhibited refined and homogeneous grains without any detectable porosity. In contrast, when the initial freezing temperature was increased, as shown in Figure 6e,f, the grain size became coarser. Moreover, under high moisture content combined with high freezing temperatures, large gas pores appeared in the microstructure. This occurred because higher freezing temperatures promote the transformation of ice-crystal bonding bridges into liquid water, generating excessive water vapor and leading to localized dry sand regions. Under these conditions, the negative pressure was unable to remove all vapor promptly, resulting in gas entrapment in the melt and the formation of macroscopic pores.

With a constant freezing temperature of −40 °C, a decrease in moisture content led to gradual coarsening of the microstructure, as seen in Figure 6g. This is because, under negative-pressure conditions, higher moisture levels improve the interfacial heat transfer coefficient (IHTC), enhancing heat extraction and refining grains. At lower moisture levels, as shown in Figure 6h, limited vapor generation allowed most gases to be evacuated effectively under negative pressure. However, the insufficient moisture is considered to have weakened the frozen sand near the melt, presumably degrading the interfacial contact and adversely affecting the surface quality and dimensional integrity of the casting.

The area fraction of porosity in each condition was quantified from EPMA images using ImageJ thresholding (Table 4). Resin sand casting without vent holes (Figure 6a) exhibited the highest porosity area fraction (~3.2%), primarily composed of gas pores. Under negative-pressure resin sand casting (Figure 6b), no significant porosity was detected (<0.1%), confirming the effectiveness of negative pressure in gas evacuation. Frozen sand casting under atmospheric conditions (Figure 6c) showed distributed micropores with an area fraction of ~1.8%, attributable to water vapor entrapment. Under optimized negative-pressure frozen sand casting (6 wt.%, −40 °C, −0.03 MPa; Figure 6d), the porosity area fraction was reduced to <0.2%, while conditions with lower moisture content (Figure 6h, 2 wt.%) or higher freezing temperature (Figure 6e, −20 °C) exhibited both micro-pores and mold-disintegration-related macro-voids, with area fractions of 0.8–2.1%.

### 3.3. Study on the Variation of Mechanical Properties

ZL205A aluminum alloy is typically used in the T6 condition to meet aerospace structural requirements; the as-cast mechanical data reported here serve primarily to evaluate solidification quality and enable process comparison, while the T6 data represent the final service properties.

#### 3.3.1. Comparison of Mechanical Properties Under Different Casting Processes

**Figure 7:**
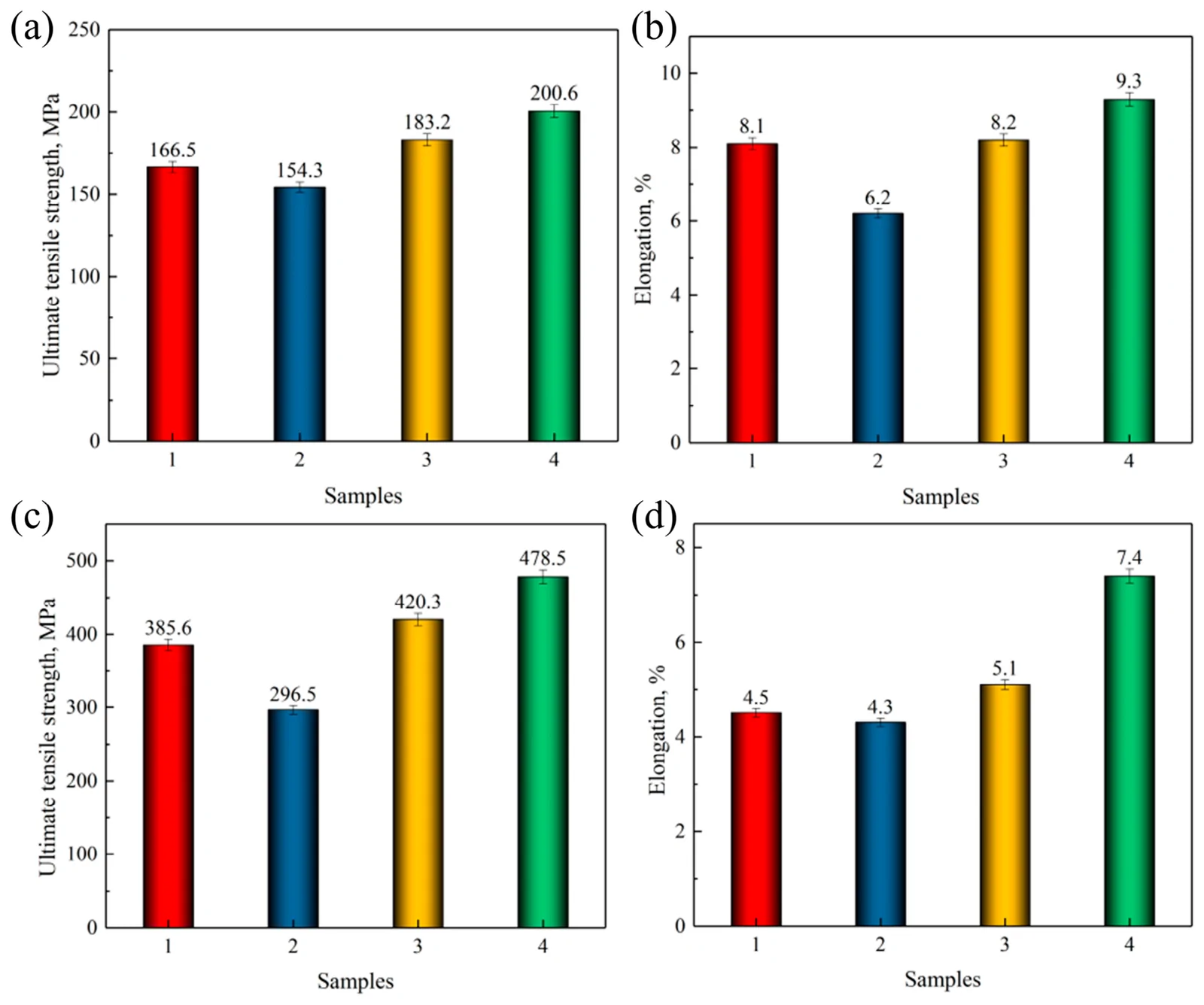
Mechanical properties of the alloy under different process conditions: (**a**) tensile strength in as-cast condition; (**b**) elongation in as-cast condition; (**c**) tensile strength after heat treatment; (**d**) elongation after heat treatment. (Note: 1 denotes resin sand, atmospheric pressure; 2 denotes resin sand and negative pressure; 3 denotes 6 wt.%, −40 °C freezing temperature, and atmospheric pressure; 4 denotes 6 wt.%, −40 °C freezing temperature, and negative-pressure cast).

Figure 7 shows the as-cast mechanical properties of the alloy under different processing conditions. For resin sand casting under atmospheric pressure, the tensile strength reached 166.5 MPa with an elongation of 8.1% in the as-cast state, and 385.6 MPa with an elongation of 4.5% after heat treatment. However, with the application of negative pressure, these values decreased to 154.3 MPa and 6.2% in the as-cast state, and 296.5 MPa and 4.3% after heat treatment. Although negative pressure enhances heat transfer between the alloy melt and the sand mold, the rapid and overall temperature rise in the resin sand mold near the melt gradually reduces the temperature difference between the alloy and the mold. As a result, although the overall cooling rate increases, a sufficiently high thermal gradient and driving force cannot be established in localized regions during the early solidification stage. This adversely affects the solidification rate, limiting microstructural refinement and leading to inferior mechanical properties.

Compared to resin sand casting under atmospheric pressure, frozen sand casting under atmospheric conditions significantly improved the mechanical properties, achieving a tensile strength of 183.2 MPa and elongation of 8.2% in the as-cast state, and 420.3 MPa and 5.1% after heat treatment. This enhancement can be attributed to the greater undercooling provided by the frozen sand mold, which promotes the formation of a denser and more uniform microstructure. Increased undercooling also facilitates heterogeneous nucleation, further refining the grains and improving the mechanical properties of the castings. The large thermal gradient rapidly increases the solidification rate, thereby refining the grain structure. The moisture in the frozen sand mold helps maintain a temperature plateau during casting, which sustains a significant temperature difference relative to the solidifying melt, supporting microstructural refinement and homogenization. Consequently, the mechanical properties are improved. However, the tendency of water vapor to penetrate the aluminum melt, leading to defects such as gas entrapment, shrinkage pores, and gas porosity, limits the extent of improvement in mechanical properties achievable with frozen sand casting.

With the application of negative pressure, the mechanical properties of the alloy castings improved significantly, achieving a tensile strength of 200.6 MPa and an elongation of 9.3% in the as-cast state, and 478.5 MPa and 7.4% after heat treatment. This enhancement is attributed to the negative-pressure environment within the frozen sand-mold cavity, which increases the melt flow velocity and facilitates the timely removal of water vapor and other gases generated at the interface between the high-temperature melt and the frozen sand. This effectively reduces defects such as gas porosity and inclusions, which are major factors contributing to the degradation of mechanical properties in castings. Furthermore, negative-pressure-assisted frozen sand casting increases the interfacial heat transfer coefficient. Combined with the low temperature of the frozen sand mold, this accelerates the solidification rate of the metal melt, resulting in grain refinement. The increased number of grain boundaries hinders crack propagation, thereby improving the strength and toughness of the castings and ultimately enhancing both solidification conditions and microstructure.

**Figure 8:**
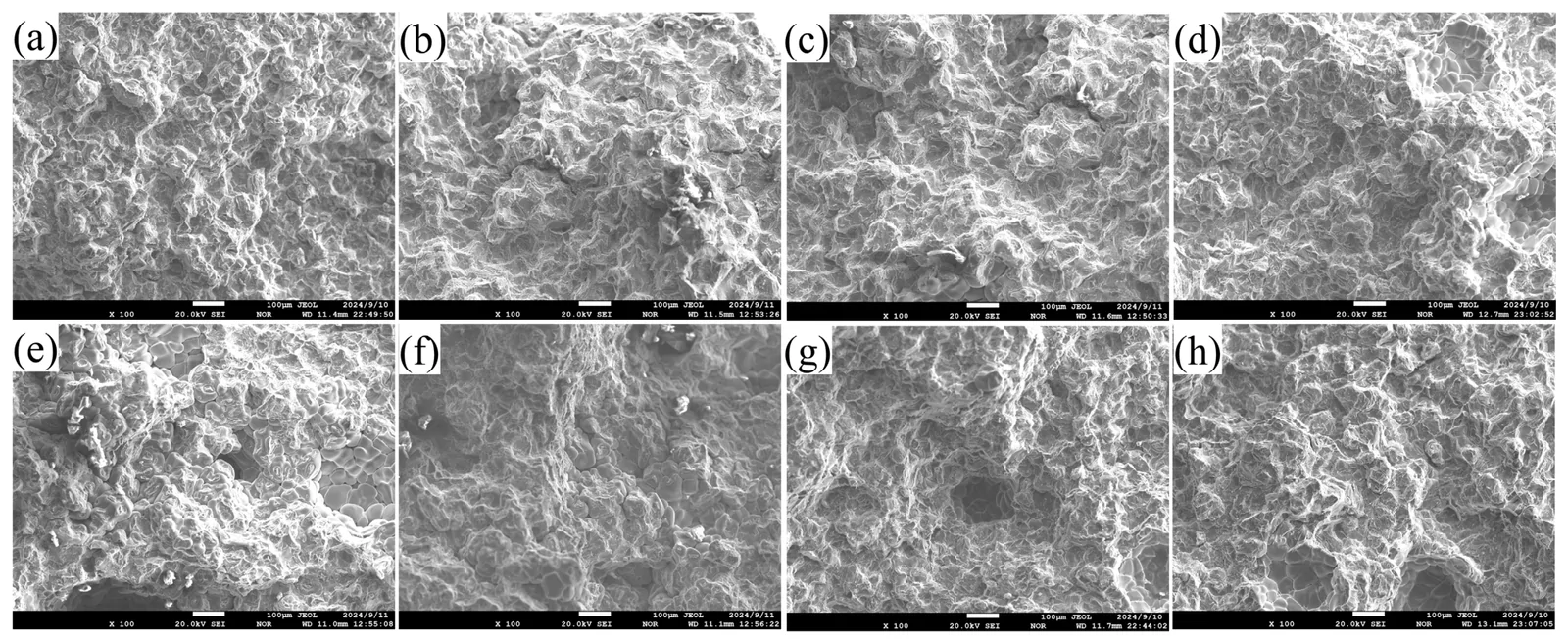
Scanning photographs of tensile fracture surfaces of castings produced by different processes. (**a**) Resin sand mold gravity casting; (**b**) resin sand mold negative-pressure casting; (**c**) 6 wt.% moisture content, −40 °C frozen sand-mold gravity casting; (**d**) 6 wt.% moisture content, −40 °C frozen sand-mold negative-pressure casting; (**e**) 6 wt.% moisture content, −20 °C frozen sand-mold negative-pressure casting; (**f**) 6 wt.% moisture content, −30 °C frozen sand-mold negative-pressure casting; (**g**) 4 wt.% moisture content, −40 °C frozen sand-mold negative-pressure casting; (**h**) 2 wt.% moisture content, −40 °C frozen sand-mold negative-pressure casting.

**Figure 9:**
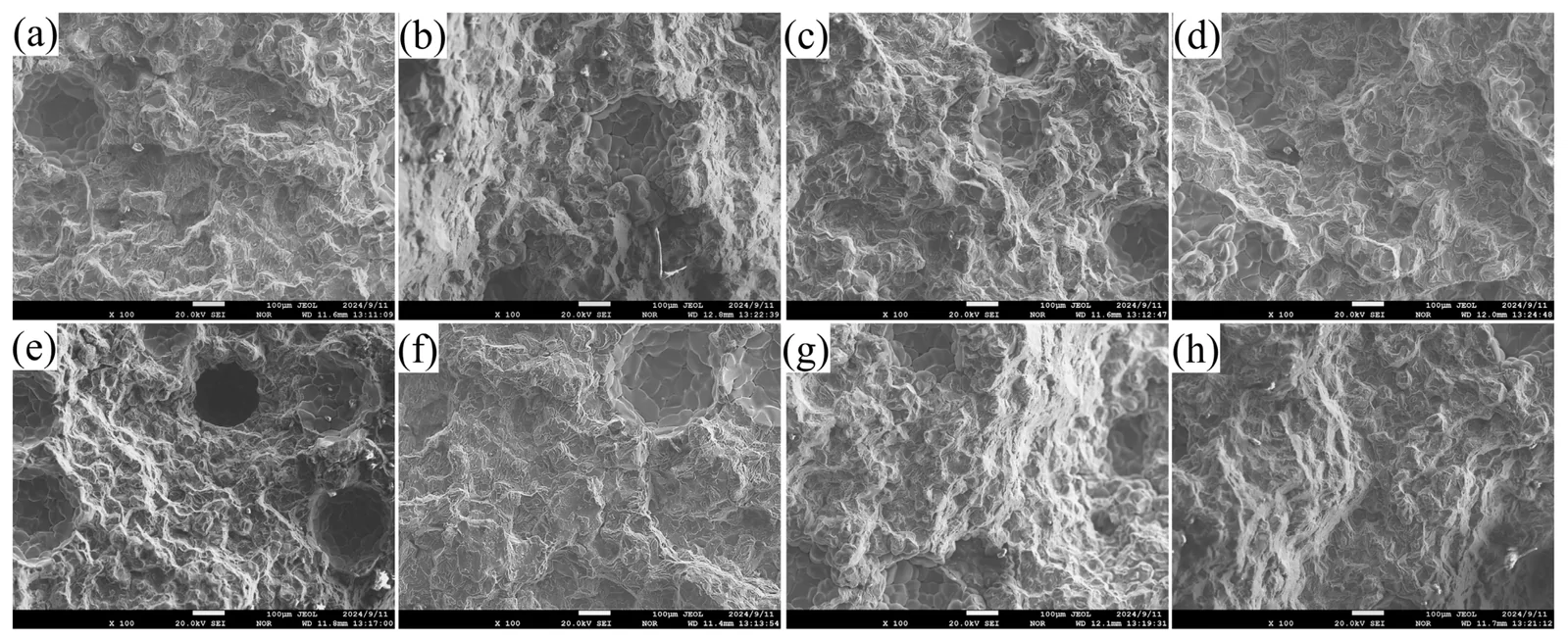
Scanning micrographs of tensile fracture surfaces after heat treatment processes. (**a**) Resin sand-mold atmospheric-pressure casting; (**b**) resin sand-mold negative-pressure casting; (**c**) 6 wt.% moisture content, −40 °C frozen sand-mold atmospheric-pressure casting; (**d**) 6 wt.% moisture content, −40 °C frozen sand-mold negative-pressure casting; (**e**) 6 wt.% moisture content, −20 °C frozen sand-mold negative-pressure casting; (**f**) 6 wt.% moisture content, −30 °C frozen sand-mold negative-pressure casting; (**g**) 4 wt.% moisture content, −40 °C frozen sand-mold negative-pressure casting; (**h**) 2 wt.% moisture content, −40 °C frozen sand-mold negative-pressure casting.

Fracture surface analysis based on the scanning electron microscopy images in Figure 8 and Figure 9 reveals that negative-pressure-assisted resin sand casting produced fracture surfaces with few dimples and the presence of inclusions and large insoluble phases, indicating inferior alloy performance under these conditions, as shown in Figure 8b and Figure 9b. Under atmospheric resin sand casting, a small number of dimples were observed, reflecting a moderate improvement in properties compared to the negative-pressure process, as seen in Figure 8a and Figure 9a. When using frozen sand casting with 6 wt.% moisture content and an initial freezing temperature of −40 °C under atmospheric pressure, the number of dimples increased, but some gas-porosity defects were detected in the fracture surface, which partially compromised the mechanical properties, as illustrated in Figure 8c and Figure 9c. In contrast, when negative-pressure-assisted frozen sand casting was employed under the same conditions (6 wt.% moisture, −40 °C initial freezing temperature, and −0.03 MPa negative pressure), the tensile fracture surface exhibited a notably high density of uniformly distributed dimples, demonstrating superior strength and toughness of the alloy castings produced by this process, as shown in Figure 8d and Figure 9d.

#### 3.3.2. Comparison of Mechanical Properties of Negative-Pressure Cast Alloy Castings with Different Frozen Sand-Mold Parameters

**Figure 10:**
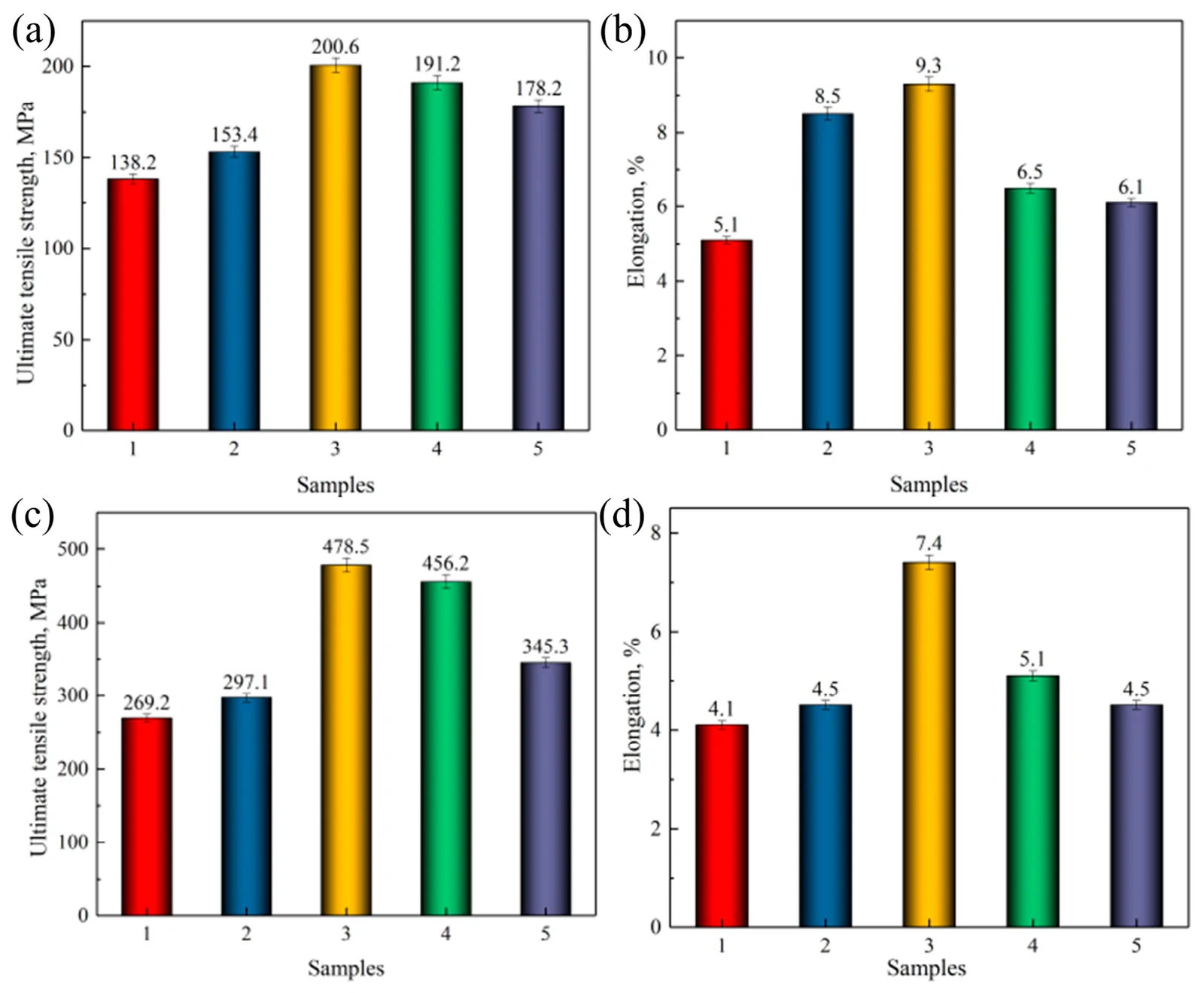
Mechanical properties of alloys under different process conditions. (**a**) Tensile strength in as-cast condition; (**b**) elongation in as-cast condition; (**c**) tensile strength after heat treatment; (**d**) elongation after heat treatment. (Note: 1 denotes 6 wt.%, −20 °C; 2 denotes 6 wt.%, −30 °C; 3 denotes 6 wt.%, −40 °C; 4 denotes 4 wt.%, −40 °C; 5 denotes 2 wt.%, −40 °C; all under negative-pressure cast).

By adjusting the moisture content of the frozen sand mold from 2 wt.% to 6 wt.% and the initial freezing temperature from −40 °C to −20 °C, the mechanical properties of negative-pressure-cast specimens under different frozen sand-mold parameters were compared, as shown in Figure 10. When the moisture content was constant at 6 wt.%, as the freezing temperature decreased from −20 °C to −40 °C, the mechanical properties of the alloy castings gradually improved. The as-cast tensile strength increased from 138.2 MPa with an elongation of 5.1%, and the heat-treated tensile strength from 269.2 MPa with an elongation of 4.1% (−20 °C), to 200.6 MPa with an elongation of 9.3% in the as-cast state, and 478.5 MPa with an elongation of 7.4% after heat treatment (−40 °C). This improvement is attributed to the lower freezing temperature, which creates a greater temperature gradient between the alloy melt and the frozen sand mold. Under negative pressure, this helps maintain a low-temperature plateau that continuously and rapidly cools the melt, increasing the solidification rate and refining the microstructure. Furthermore, the negative-pressure environment rapidly extracts water vapor, minimizing its adverse effects on the alloy melt. As a result, both the surface quality and mechanical properties of the castings are enhanced.

As also observed in Figure 10, under the same freezing temperature condition, a decrease in moisture content leads to a reduction in mechanical properties. For example, at a constant freezing temperature of −40 °C, as the moisture content decreased from 6 wt.% to 2 wt.%, the mechanical properties declined gradually: the as-cast tensile strength decreased from 200.6 MPa with 9.3% elongation and the heat-treated tensile strength from 478.5 MPa with 7.4% elongation (6 wt.%) to 178.2 MPa with 6.1% elongation in the as-cast state and 345.3 MPa with 4.5% elongation after heat treatment (2 wt.%). This decline occurs because, at lower moisture content and lower freezing temperature, the high interfacial heat transfer coefficient causes rapid heating of the frozen sand in contact with the melt. The combined effect of negative pressure quickly removes water vapor, leading to dry sand conditions at the mold–metal interface. This can cause mold deformation, collapse, or even disintegration. Although the negative-pressure chamber provides some support to the mold, the surface quality of the casting is still significantly compromised. Although the accelerated heat transfer promotes localized microstructural refinement and some improvement in properties, making it superior to other processes, the degraded surface quality can result in non-conforming products. Additionally, sand particles may become incorporated into the melt, leading to inclusion defects in the castings.

Therefore, for negative-pressure-assisted frozen sand casting, both the freezing temperature and moisture content critically influence mold performance. It is essential to maintain a relatively high moisture content and a low freezing temperature to ensure both the surface quality of the alloy castings and the enhancement of their mechanical properties.

Fractographic analysis based on the scanning electron microscopy images in Figure 8 and Figure 9 supports these conclusions. When using a frozen sand mold with 6 wt.% moisture content and an initial freezing temperature of −40 °C under a negative pressure of −0.03 MPa, the tensile fracture surface exhibited a high density of uniformly distributed dimples, indicating superior strength and toughness of the alloy castings, as shown in Figure 8d and Figure 9d. At a constant freezing temperature of −40 °C, a decrease in moisture content resulted in a gradual reduction in the number of dimples and the appearance of insoluble precipitates, which adversely affected the mechanical properties, as illustrated in Figure 8g,h and Figure 9g,h. Similarly, at a constant moisture content of 6 wt.%, an increase in the freezing temperature led to a noticeable decrease in dimple density and the emergence of coarse inclusions, as shown in Figure 8e,f and Figure 9e,f. This is attributed to the higher temperature promoting the disintegration of the sand mold near the melt, allowing foreign particles to enter the solidifying alloy and significantly degrading its mechanical properties.

### 3.4. Validation with Typical Components

**Figure 11:**
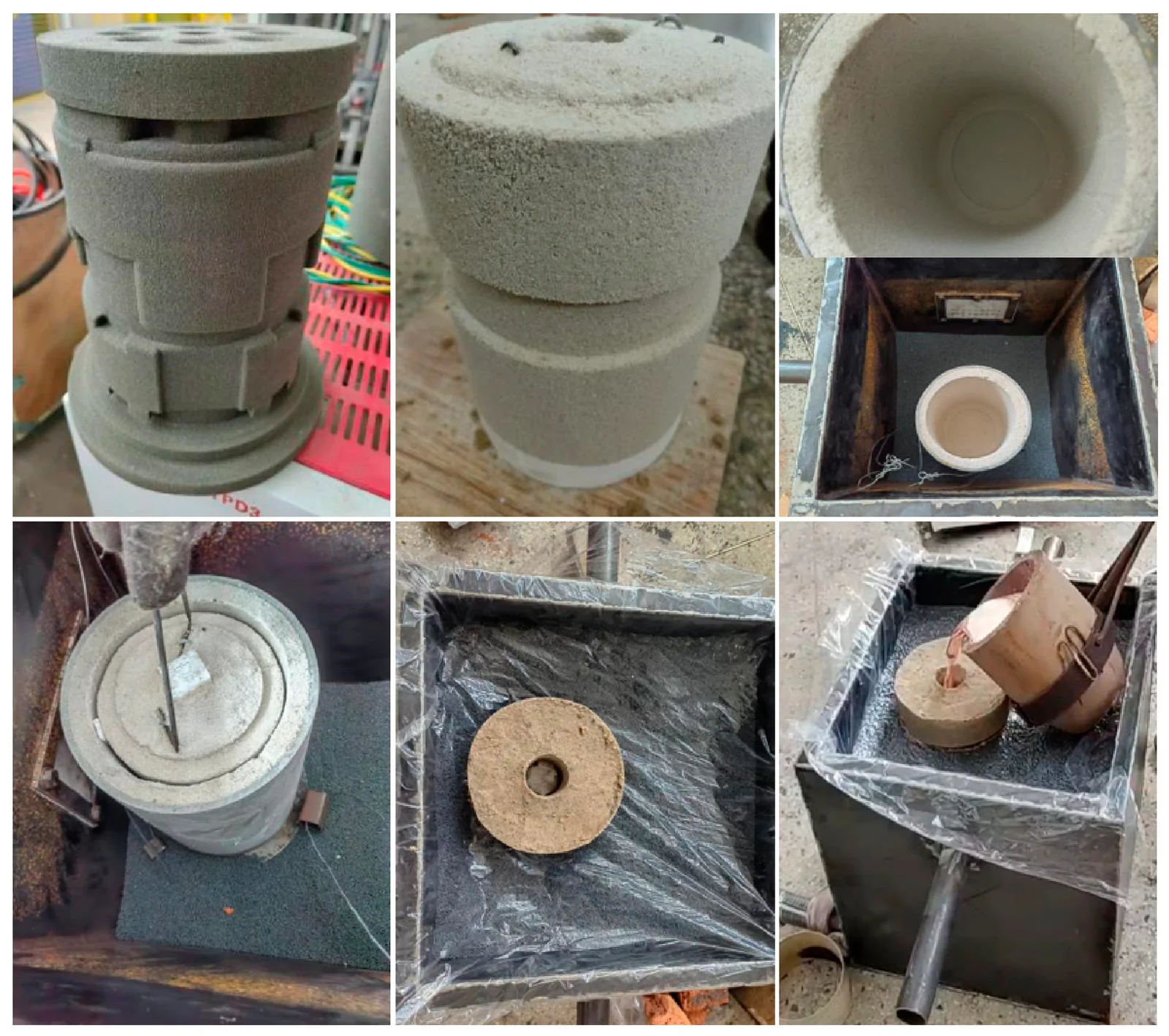
Preparation of different simulated sand molds and negative-pressure casting of frozen sand molds.

The thin-walled cabinet simulators with an outer diameter of 180 mm, a height of 300 mm, and a minimum wall thickness of 3 mm were designed for experimental validation. The ZL205A cast aluminum alloy was used, and five different processes were employed to form the thin-walled cabinet components, including the resin sand casting under atmospheric pressure, frozen sand casting (6 wt.% moisture, −40 °C) under atmospheric pressure, negative-pressure-assisted frozen sand casting (2 wt.% moisture, −40 °C, −0.03 MPa), negative-pressure-assisted frozen sand casting (4 wt.% moisture, −40 °C, −0.03 MPa), and negative-pressure-assisted frozen sand casting (6 wt.% moisture, −40 °C, −0.03 MPa). The resin sand molds and frozen sand molds used in the study are shown in Figure 11. A bottom gating system was adopted, with a horizontal runner incorporated at the base. The thin-walled cabinet components were cast using a combination of outer sand molds and cores.

**Figure 12:**
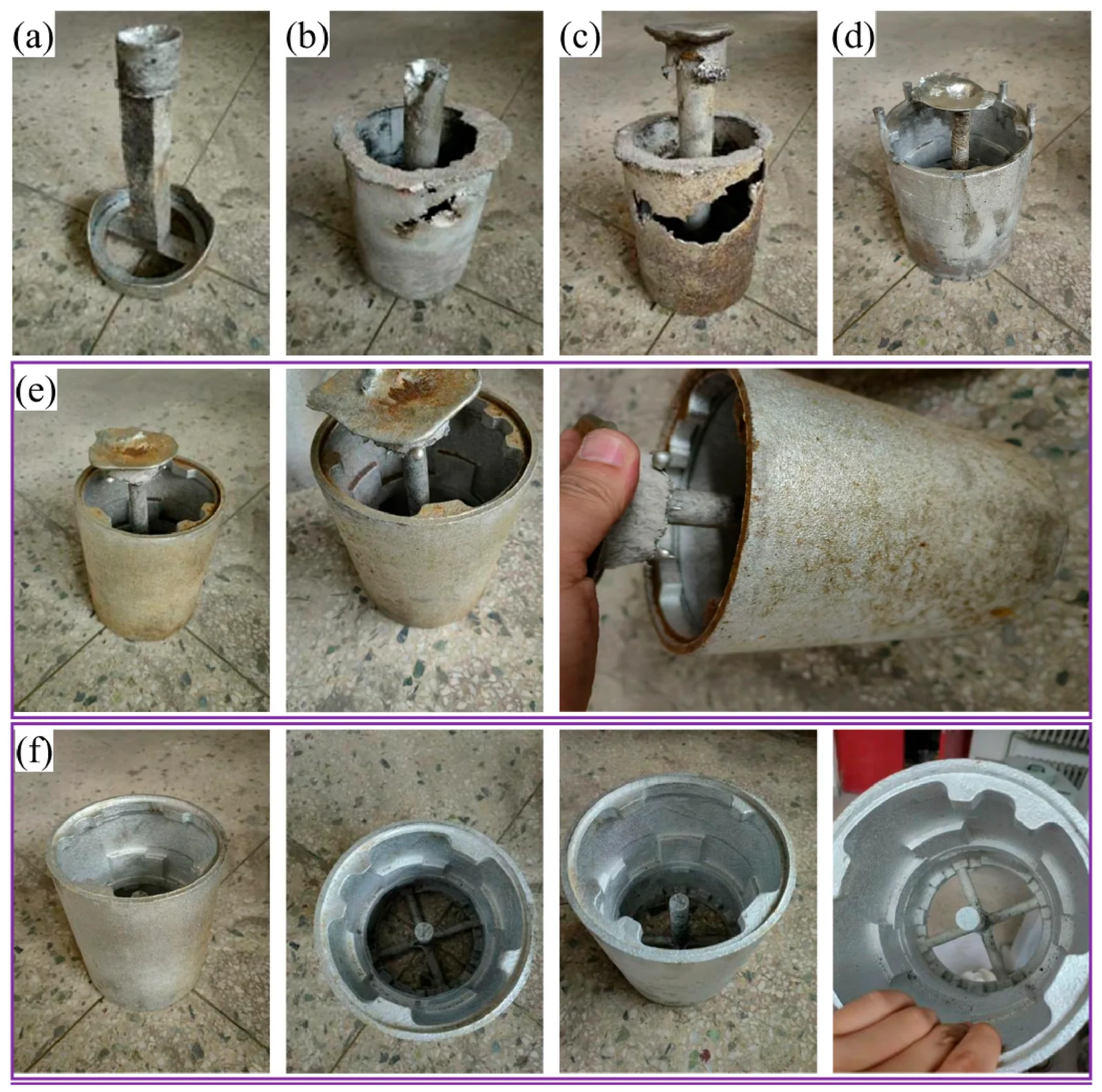
ZL205A aluminum alloy cabin parts under different processes. (**a**) Resin sand-mold normal-pressure casting; (**b**) water content 6 wt.%, −40 °C frozen sand-mold normal-pressure casting; (**c**) water content 2 wt.%, −40 °C frozen sand-mold −0.03 MPa negative-pressure casting; (**d**) water content 4 wt.%, −40 °C frozen sand-mold −0.03 MPa negative-pressure casting; (**e**,**f**) 6 wt.% moisture content, −40 °C frozen sand-mold −0.03 MPa negative-pressure casting for small-sized cabin components.

The cast specimens produced by different processes are shown in Figure 12. It could be observed that producing thin-walled castings using resin sand casting was particularly challenging due to the poor fluidity of ZL205A aluminum alloy melt and its high susceptibility to defects such as shrinkage porosity and cavities. As a result, incomplete filling and insufficient feeding occurred during resin sand casting, as illustrated in Figure 12a. When frozen sand casting under atmospheric pressure was employed, as shown in Figure 12b, the mold enhanced the fluidity and filling capability of the alloy melt to some extent. This improvement was attributed to the formation of a slight air gap between the melt and the mold caused by water vapor generation, which reduced friction and facilitated melt flow. Although this represented a noticeable improvement over resin sand casting, the accelerated solidification rate induced by the frozen sand mold shortened the filling time. Consequently, the process remained insufficient for effectively casting thin-walled ZL205A aluminum alloy components with a wide solidification interval.

When negative-pressure-assisted casting was applied using a frozen sand mold with 2 wt.% moisture content and an initial freezing temperature of −40 °C, as shown in Figure 12c, it was observed that although the typical casting exhibited improved filling capability compared to resin sand casting, relatively large pores formed in contrast to atmospheric casting. This was attributed to the low freezing temperature, which resulted in a high interfacial heat transfer coefficient, while the low moisture content is considered to have weakened the sand mold near the interface, presumably degrading the interfacial contact and leading to poor casting formation. When the moisture content of the frozen sand mold was increased to 4 wt.% for negative-pressure-assisted casting, a significant improvement in the surface quality of the castings became evident, as shown in Figure 12d. On further employing a frozen sand mold with 6 wt.% moisture content and −40 °C under negative-pressure conditions, as illustrated in Figure 12e,f, both the inner and outer surfaces of the cabinet component showed remarkable enhancement, with complete filling and satisfactory macroscopic and dimensional quality, thereby meeting product qualification standards. This improvement was due to the fact that the combination of 6 wt.% moisture content and −40 °C freezing temperature in negative-pressure-assisted casting not only promoted the filling capacity of the alloy melt but also reduced defects such as gas porosity, shrinkage cavities, and micro-shrinkage. Moreover, under these conditions, the frozen sand mold maintained both high interfacial heat-transfer efficiency and sufficient mold strength, preventing any deformation or collapse during solidification. To further assess dimensional accuracy, the wall thickness of the fabricated cabinet component was measured at 12 uniformly distributed locations using a vernier caliper. The measured wall thickness ranged from 3.0 to 3.4 mm (nominal: 3.0 mm), with a maximum deviation of +0.4 mm, indicating satisfactory dimensional fidelity of the negative-pressure frozen sand-casting process for thin-walled structures. Macroscopic visual inspection and cross-sectional examination (Figure 12e,f) confirmed the absence of cold shuts, incomplete filling, or surface porosity. Systematic non-destructive testing (e.g., X-ray CT) of the component is planned as a follow-up investigation to provide volumetric defect statistics.

### 3.5. Validation of the Inverse IHTC Results

To validate the IHTC values obtained by the inverse method, the measured mold-side temperatures were compared with the results of a forward heat conduction simulation of the frozen sand mold. The one-dimensional transient heat conduction equation was solved by an explicit finite-difference scheme along the direction normal to the melt–mold interface, over the mold thickness of 80 mm. At the interface, the heat flux was prescribed as q(t) = f_v_·h(t)·[*T_m_*(*t*) − *T_s_*(*t*)], where *h*(*t*) is the inversely determined IHTC (Figure 2 and Table 3), *T_m_*(*t*) is the measured melt temperature, *T_s_*(*t*) is the computed mold surface temperature, and f_v_ is the fraction of the interfacial heat flow that is conducted into the sand matrix behind the thermocouple positions, the remainder being consumed by the evaporation of moisture and the escape of vapor from the interfacial region, as discussed in Section 3.1; f_v_ was calibrated as a single effective parameter (f_v_ = 0.22) common to both validated cases. The thermo-physical properties of the frozen sand were described by an effective thermal conductivity of 1.8 W/(m·K), within the range reported for frozen sand molds [9], a density of 1550 kg/m^3^, and an apparent specific heat capacity of 850 J/(kg·K) that additionally distributes the latent heat of ice melting around 0 °C and the latent heat of moisture evaporation over the interval of 40–140 °C, following the measured plateau behavior of the mold temperatures; the nominal moisture content of 6 wt.% was used in the latent-heat terms.

**Figure 13:**
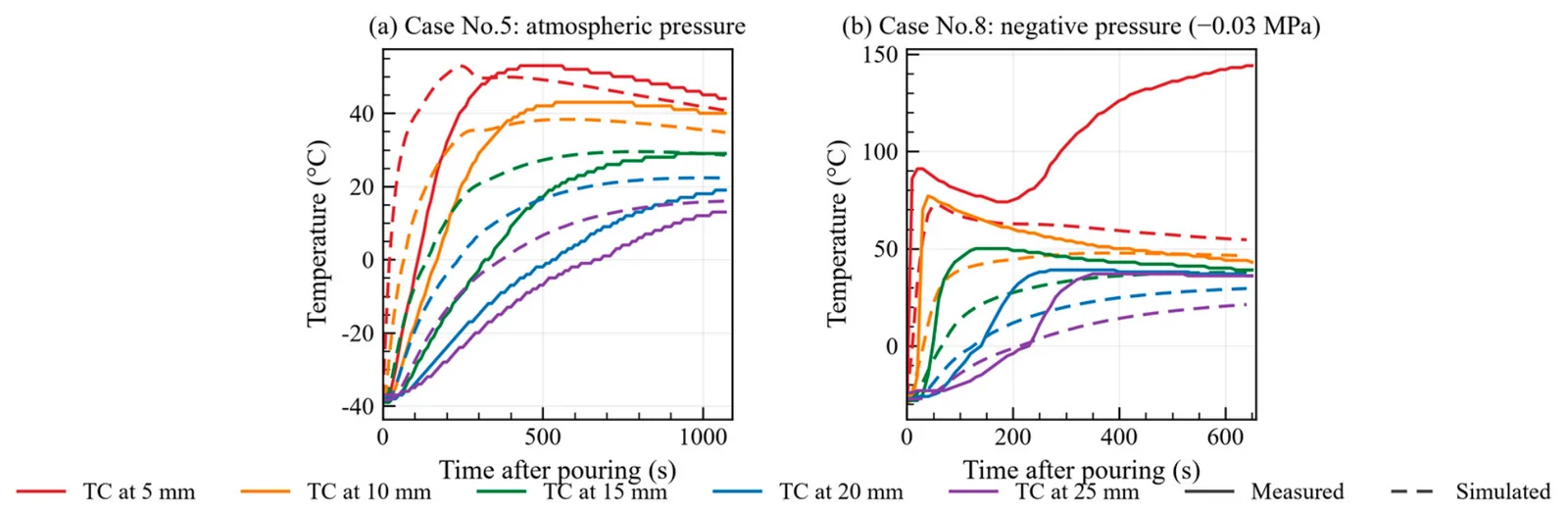
Comparison of measured (solid lines) and simulated (dashed lines) heating curves in the frozen sand mold at 5, 10, 15, 20, and 25 mm from the melt–mold interface. (**a**) Atmospheric frozen sand casting (No. 5, 6 wt.%, −40 °C); (**b**) negative-pressure frozen sand casting (No. 8, 6 wt.%, −40 °C, −0.03 MPa).

Figure 13 compares the simulated and measured heating curves at thermocouple positions of 5, 10, 15, 20, and 25 mm from the melt–mold interface for the atmospheric case (No. 5) and the negative-pressure case (No. 8), i.e., the two most relevant conditions identified in Table 3. Using the same set of effective mold parameters and the same calibrated f_v_ for both cases, with *h*(*t*) as the only case-specific input, the simulation reproduces the measured temperature histories, including the initial rapid heating near the interface, the intermediate inflections associated with ice melting and moisture evaporation, and the later gradual temperature rise: the average root-mean-square deviation between the simulated and measured curves is 13.7 °C for No. 5 (17.0, 12.2, 14.0, 14.6, and 10.8 °C at the five positions) and 24.2 °C for No. 8 (56.9, 19.8, 15.8, 12.6, and 15.8 °C). The larger deviation at the position closest to the interface under negative pressure is attributed to the steep local temperature gradient, which amplifies the effect of the ±1 mm thermocouple position uncertainty. This agreement indicates that the inversely determined IHTC values constitute physically consistent boundary conditions for the mold-side heat conduction problem and supports the quantitative comparison between the process conditions in Table 3.

## 4. Conclusions

This study investigated the temperature evolution behavior of ZL205A aluminum alloy with a wide solidification interval under different casting processes, focusing on the interaction between the alloy melt and frozen sand molds. The main conclusions are summarized as follows:

(1) Negative pressure increased the IHTC between the alloy melt and both resin-sand and frozen-sand molds. The active-period average IHTC rose from 82 W/(m^2^·K) for atmospheric resin sand to 87 W/(m^2^·K) under negative pressure, and from 84 W/(m^2^·K) for atmospheric frozen sand (6 wt.%, −40 °C) to 236 W/(m^2^·K) under negative pressure, a 2.8-fold enhancement arising from interfacial vapor removal and the sustained thermal gradient. Increasing moisture content raised the plateau temperature of frozen sand casting.

(2) Negative pressure established a pronounced temperature-gradient channel within the frozen sand mold: the region contacting the melt heated up rapidly while the outer mold remained cold, which enlarged the thermal driving force and accelerated solidification. Under negative pressure, higher moisture content and lower initial freezing temperature produced a higher IHTC and a faster solidification rate, with a peak IHTC of 1250 W/(m^2^·K) at 6 wt.% and −40 °C.

(3) Negative-pressure frozen sand casting (6 wt.%, −40 °C, −0.03 MPa) refined the average grain size from 95 μm to 58 μm and reduced the porosity area fraction from 3.2% to below 0.2%, achieving 200.6 MPa with 9.3% elongation in the as-cast state and 478.5 MPa with 7.4% after T6 treatment. The mechanism involves three coupled effects: vapor extraction enlarges the solid–solid contact area, the enhanced IHTC raises the solidification undercooling and refines the grains, and gas-induced porosity is concurrently suppressed.

(4) Using the optimized parameters, a thin-walled cabin component with successful filling and satisfactory macroscopic and dimensional quality (Ø180 mm × 300 mm, measured wall thickness 3.0–3.4 mm, maximum deviation +0.4 mm) was successfully fabricated. The reported IHTC values are geometry-dependent boundary conditions; extending them to castings with larger cross-sections or greater weight requires re-evaluation of the thermal-mass and solidification-time effects.

## Figures and Tables

**Table 1:** Chemical composition of ZL205A aluminum alloy (wt.%).

Element	Cu	Mn	Ti	Cd	V	Zr	B	Al
Content (wt.%)	4.6–5.3	0.3–0.5	0.15–0.35	0.15–0.25	0.05–0.3	≤0.2	≤0.01	Bal.

**Table 2:** Different process parameters.

No.	Material	Process	Freezing Temperature, °C	Water Content, wt.%
1	Resin sand mold	Normal pressure	-	-
2	Resin sand mold	Negative pressure, −0.03 MPa	-	-
3	Frozen sand mold	Normal pressure	−40	2
4	Frozen sand mold	Normal pressure	−40	4
5	Frozen sand mold	Normal pressure	−40	6
6	Frozen sand mold	Negative pressure, −0.03 MPa	−40	2
7	Frozen sand mold	Negative pressure, −0.03 MPa	−40	4
8	Frozen sand mold	Negative pressure, −0.03 MPa	−40	6
9	Frozen sand mold	Negative pressure, −0.03 MPa	−30	2
10	Frozen sand mold	Negative pressure, −0.03 MPa	−30	4
11	Frozen sand mold	Negative pressure, −0.03 MPa	−30	6
12	Frozen sand mold	Negative pressure, −0.03 MPa	−20	2
13	Frozen sand mold	Negative pressure, −0.03 MPa	−20	4
14	Frozen sand mold	Negative pressure, −0.03 MPa	−20	6

**Table 3:** Summary of calculated IHTC for all 14 experimental conditions.

No.	Condition	Peak h (W/(m^2^ K))	Early-Stage avg. h (W/(m^2^ K))	Active-Period avg. h (W/(m^2^ K))
1	Resin sand, atmospheric	4969	151	82
2	Resin sand, −0.03 MPa	4675	162	87
3	Frozen, −40 °C, 2 wt.%, atm.	412	118	79
4	Frozen, −40 °C, 4 wt.%, atm.	437	129	81
5	Frozen, −40 °C, 6 wt.%, atm.	458	137	84
6	Frozen, −40 °C, 2 wt.%, −0.03 MPa	705	245	131
7	Frozen, −40 °C, 4 wt.%, −0.03 MPa	892	328	125
8	Frozen, −40 °C, 6 wt.%, −0.03 MPa	1250	837	236
9	Frozen, −30 °C, 2 wt.%, −0.03 MPa	685	232	128
10	Frozen, −30 °C, 4 wt.%, −0.03 MPa	815	298	138
11	Frozen, −30 °C, 6 wt.%, −0.03 MPa	1085	612	189
12	Frozen, −20 °C, 2 wt.%, −0.03 MPa	565	192	112
13	Frozen, −20 °C, 4 wt.%, −0.03 MPa	725	265	125
14	Frozen, −20 °C, 6 wt.%, −0.03 MPa	935	476	120

**Table 4:** Quantitative grain size and porosity area fraction for representative conditions.

Condition	Grain Size (um)	Porosity Area Fraction (%)
Resin sand, atmospheric	~95	~3.2
Resin sand, −0.03 MPa	~105	<0.1
Frozen, 6 wt.%, −40 °C, atm.	~70	~1.8
Frozen, 6 wt.%, −40 °C, −0.03 MPa	~58	<0.2
Frozen, 6 wt.%, −20 °C, −0.03 MPa	~93	~2.1
Frozen, 6 wt.%, −30 °C, −0.03 MPa	~78	~1.3
Frozen, 4 wt.%, −40 °C, −0.03 MPa	~80	~1.0
Frozen, 2 wt.%, −40 °C, −0.03 MPa	~85	~0.8

## Data Availability

The original contributions presented in this study are included in the article. Further inquiries can be directed to the corresponding authors.
